# Recent Advances in Dual COX/LOX Inhibitor Design (2020–2024): Establishing “*The Rule of Four for Inflammation*”

**DOI:** 10.3390/life16010163

**Published:** 2026-01-19

**Authors:** Filippos Panteleimon Chatzipieris, Errikos Petsas, George Lambrinidis, Stamatia Vassiliou, Christos T. Chasapis

**Affiliations:** 1 Laboratory of Organic Chemistry, Department of Chemistry, National and Kapodistrian University of Athens, 15771 Athens, Greece; fchatzip@chem.uoa.gr (F.P.C.); errpets@chem.uoa.gr (E.P.); svassiliou@chem.uoa.gr (S.V.); 2 Division of Medicinal Chemistry, Department of Pharmacy, School of Health Sciences, National & Kapodistrian University of Athens, 15771 Athens, Greece; lamprinidis@pharm.uoa.gr

**Keywords:** arachidonic acid (AA), inflammation, cyclooxygenase-2 (COX-2), 5-lipoxygenase (5-LOX), dual COX-2/5-LOX inhibitors, structure–activity relationship (SAR), the rule of four for inflammation, MATLAB, pharmacophore model

## Abstract

The arachidonic acid pathway plays a pivotal role in the biosynthesis of important inflammatory and signal transducing agents such as prostaglandins, leukotrienes and thromboxanes. When this pathway is deregulated, it leads to pathological conditions such as cardiovascular diseases, metabolic diseases, and cancer. Two key enzymes of the pathway are cyclooxygenases (COXs) and lipoxygenases (LOXs), which are responsible for the production of prostaglandins and leukotrienes, respectively. Consequently, these enzymes have long been recognized as key therapeutic targets for the treatment and management of inflammatory disorders and other pathological conditions associated with inflammation. In this review, we describe the new evidence over the last 4 years regarding the arachidonic acid pathway. Moreover, we will pay attention to the structure and function of the COX-2 and 5-LOX enzymes and their role in inflammation, as well as define their active sites. Later, we will discuss the most potent, dual inhibitors of COX-2 and 5-LOX enzymes, based on in vitro and in vivo experiments, from 2020–2024. Structure–activity relationship (SAR) analysis of these compounds revealed four key structural features required for potent dual inhibition of cyclooxygenase-2 (COX-2) and 5-lipoxygenase (5-LOX). We refer to these criteria as “*The Rule of Four for Inflammation*”.

## 1. Introduction

Inflammation is a complex biological response to harmful stimuli, like injury or infection, which involves a cascade of events aimed at protecting the body. It starts with tissue damage, leading to the release of chemical mediators and the activation of various immune cells [1]. The main enzymes which metabolize arachidonic acid (AA) are cyclooxygenases (COXs), resulting in the formation of prostaglandins (PGs) and thromboxanes, and lipoxygenases (LOXs), resulting in the formation of leukotrienes (LTs) [2]. PGs act as vasodilators [3], thromboxanes act as vasoconstrictors [4], and LTs trigger contractions in the smooth muscles [5]. The AA pathway is a crucial regulator in cardiovascular function [6], tumor development [7], and a variety of inflammatory disorders, including asthma and arthritis [8,9,10]. AA metabolism is involved not only in cell differentiation, tissue development, and organ function but also in the progression of diseases, such as hepatic fibrosis, neurodegeneration, obesity, diabetes [11]. There is also a correlation between hypertension and inflammation [12,13,14] and it has become evident in the last decade that inflammation plays a pivotal role in the pathogenesis and progression of essential hypertension [15,16,17,18,19]. Inflammation has also been linked as a risk factor for neurodegenerative diseases, such as Alzheimer’s disease (AD) [20]. Since the inhibition of the angiotensin II type 1 (AT1) receptor can help delay the onset and progression of AD [21,22], creation of new drug entities which can combine anti-inflammatory and anti-hypertensive effects, as well as lower the amyloid beta (Aβ) plaques on the brain, could be the next step in AD therapy.

Thus, the focus of this review will be on the study of multi-directed ligands (MTDLs) for the dual inhibition of COX/LOX, addressing two inflammatory pathways in one go. For the purposes of this review, the inhibition of specific enzymes COX-2 and 5-LOX will be studied. Specifically, we will examine the newly developed dual inhibitors from 2020 to 2024 to achieve the aforementioned goals.

**Figure 1:**
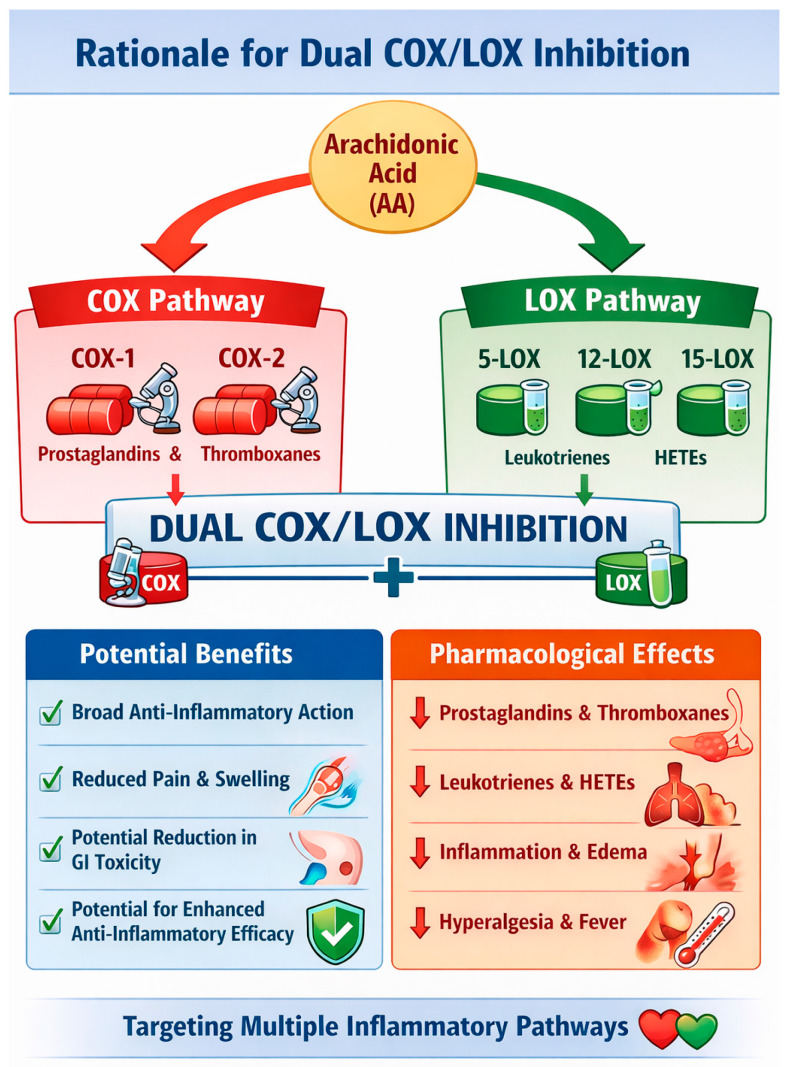
Illustration of the mechanistic rationale for dual COX/LOX inhibition. The figure summarizes intended pharmacological effects based on arachidonic acid (AA) metabolism and does not imply uniform clinical efficacy or safety across all dual inhibitors.

The reasoning behind selective COX-2 inhibition is due to the fact that COX-1 catalyzes the formation of cytoprotective PGs in thrombocytes, vascular endothelium, stomach mucosa, kidneys, pancreas, Langerhans islets, seminal vesicles, and brain. Thus, inhibition of COX-1 can lead to gastrointestinal erosions, and renal and hepatic insufficiency [23]. Simultaneously, 5-LOX is the key enzyme for producing pro-inflammatory LTs, which are central to allergic and inflammatory responses [24] and thus it can be a very promising drug target (Figure 1).

Targeting several pathways at once has proven effective in improving drug performance and combating resistance, particularly in complex diseases. From Alzheimer’s to cancer, more and more data show that most of the publicly known diseases are actually multifactorial. Although the ‘one molecule–one target’ strategy has significantly improved global health outcomes, it is now considered outdated. Attention should instead focus on developing Multi-Target Directed Ligands (MTDLs), such as dual inhibitors, which are rationally designed to act on multiple targets and are increasingly recognized as a promising approach in modern drug discovery [21,25,26,27,28].

It should be noted that dual inhibition of COX and LOX may result in metabolic shunting of AA toward the cytochrome P450 pathway, potentially enhancing the formation of biologically active HETEs and EETs. These metabolites have been implicated in vasodilatation, angiogenesis, renal sodium handling, and cellular proliferation, raising the possibility that chronic COX/LOX dual inhibition could produce unintended cardiovascular, renal, or pro-angiogenic effects that warrant careful evaluation [29,30,31]. However, since EETs have vasodilatory, cardioprotective, and anti-inflammatory activities while HETEs can mediate androgen-induced hypertension and cause vasoconstriction of vascular smooth muscle cells and vascular inflammation [2], it is difficult to hypothesize the exact adverse effects that may be introduced. Nonetheless, care should be taken in the administration regimen of these drugs.

Given the central role of COX-1-derived thromboxane A_2_ (TXA_2_) and LOX-derived eicosanoids in platelet activation, dual COX/LOX inhibitors may exert significant effects on platelet aggregation, thrombus formation, and platelet–leukocyte interactions. Experimental evidence indicates that concomitant inhibition of COX and LOX pathways can attenuate thrombus growth more effectively than COX inhibition alone, potentially offering enhanced antithrombotic efficacy. However, such effects may also influence hemostatic balance, underscoring the importance of carefully evaluating platelet function and bleeding risk during the development of novel dual COX/LOX inhibitors [32,33].

Early dual inhibitors, such as darbufelone and licofelone, showed toxicity and efficacy imbalances, cardiovascular risks, and pharmacokinetic limitations. These outcomes highlight that while dual COX/LOX inhibition remains a promising strategy, careful optimization of efficacy, safety, and pharmacokinetic properties is essential for clinical success [34].

Although early clinical candidates such as licofelone did not achieve regulatory approval [35], these outcomes did not invalidate the dual COX/LOX concept but rather revealed critical limitations related to pathway balance, metabolic shunting, and safety optimization. Advances in structural biology, eicosanoid profiling, and systems pharmacology have since reshaped the design of dual COX/LOX inhibitors, shifting the field from nonspecific enzyme blockade toward rational, pathway-biased modulation. This evolution explains the sustained and increasing research activity observed from 2020 onward. The persistence of dual COX/LOX research, reflects a broader shift in drug discovery philosophy, where earlier clinical failures are leveraged to refine, rather than abandon, multi-target therapeutic strategies.

We will start our discussion by deciphering the AA pathway, as well as introducing novel information related to it. The key enzymes involved in the metabolism of AA will be studied from a structural perspective and their active sites will be established. Finally, as we have mentioned, dual inhibitors of COX-2 and 5-LOX enzymes will be reviewed for the period of 2020–2024.

Analysis of the structure–activity relationships (SARs) of these compounds revealed four key structural features required for potent dual inhibition of cyclooxygenase-2 (COX-2) and 5-lipoxygenase (5-LOX). We refer to these criteria as “*The Rule of Four for Inflammation*”. First, an effective inhibitor typically possesses an extended carbon framework of approximately 18 carbon atoms. Second, the presence of several rotatable bonds—allowing greater conformational flexibility—is essential. Third, the molecule should incorporate an expanded aromatic system composed of multiple benzene rings. Finally, the inclusion of a five-membered heterocycle (pyrazole-like), directly linked at its 4-position to a benzenesulfonamide moiety and bearing either an amine or methyl substituent at the 5-position, enhances binding interactions within the active sites of both enzymes, thereby improving inhibitory potency.

### 1.1. The Role of the Arachidonic Acid Pathway in Regulating Inflammation

**Figure 2:**
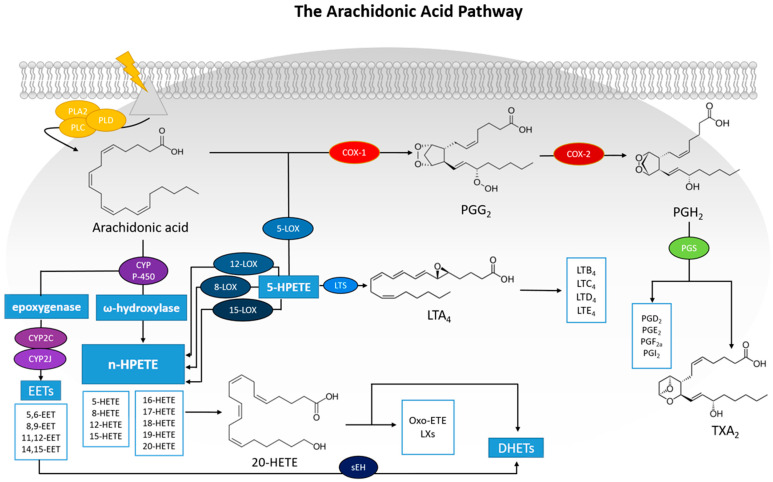
The AA pathway is of major importance in the regulation of inflammation. When this pathway is deregulated pathological conditions such as cardiovascular diseases, metabolic diseases and cancer are established. AA can be metabolized directly by three types of enzymes, cyclooxygenases (COXs), lipoxygenases (LOXs) and cytochrome P450, into prostaglandins (PGs) and thromboxane A_2_ (TXA_2_), leukotrienes (LTs) and hydroxyeicosatetraenoic acids (HETEs), and epoxyeicosatrienoic acids (EETs), respectively. EETs are primarily broken down by soluble epoxide hydrolase (sEH), which converts them into their corresponding diols, known as dihydroxyeicosatrienoic acids (DHETs). COX-1 incorporates two oxygen molecules to generate the 1,2-dioxane ring and a peroxide group, producing prostaglandin G_2_ (PGG_2_). COX-2 then converts this peroxide group into a secondary alcohol, yielding prostaglandin H_2_ (PGH_2_). Downstream prostaglandin synthases (PGS) convert PGH_2_ to specific PGs, including PGE_2_, PGI_2_, PGD_2_, PGF_2_, and TXA_2_. 5-LOX catalyzes the oxidation of AA to form 5-hydroperoxyeicosatetraenoate (5-HPETE). This is then transformed into leukotriene A_4_ (LTA_4_) by leukotriene synthase (LTS).

The ω-6 polyunsaturated fatty acid (PUFA), arachidonic acid (AA), is a very important biological modulator which can be metabolized directly by three types of enzymes; COXs, LOXs and cytochrome P450 [36]. This results in the creation of a lot of well-known fatty acid biochemical mediators. The COX enzymes metabolize AA to PGs and thromboxane A_2_ (TXA_2_), which belong to the family of prostanoids [11,37]. This fatty acid is released from the plasma membranes through the combined work of phospholipase enzymes’ A2 (PLA2), C (PLC) and D (PLD) (Figure 2). Subsequently, AA is metabolized by COX enzymes to PGG_2_ and PGH_2_. These precursor molecules are then converted to PGs through the action of PG synthases (PGS) [38].

Two main COX isoforms have been identified. COX-1 is expressed continuously in most tissues and is the primary producer of prostanoids involved in essential physiological maintenance. COX-2 (PTGS2), in contrast, is upregulated in response to inflammatory signals, hormones, and growth factors, and is typically viewed as the principal enzyme driving prostanoid synthesis during inflammation and in proliferative disorders, including cancer. Still, the distinction between them is not absolute, since both isoforms can generate prostanoids required for autoregulation and homeostasis, and both may participate in prostanoid production under inflammatory conditions [39,40].

The LOX pathway became the second major eicosanoid and inflammation-related pathway to be exploited for therapeutic intervention. The enzymes generate LTs [41]. Arachidonate 5-LOX (ALOX5) inhibitors and leukotriene receptor blockers have been developed as therapies for asthma and seasonal allergic conditions [42,43]. As research uncovers new receptors and metabolites, the COX and LOX eicosanoid pathways are gaining prominence as therapeutic targets, with their involvement in numerous diseases becoming increasingly well understood [44].

The third pathway through which AA can be metabolized is via the work of cytochrome P450 (CYP). The CYP enzyme family includes many subclasses, but in the context of AA metabolism, the ω-hydroxylase and epoxygenase activities are the most significant [45]. Nonetheless, several CYP isoforms display overlapping hydroxylase and epoxygenase functions, enabling them to produce a diverse mixture of metabolites. The ω-hydroxylase activity of CYP enzymes transforms AA into hydroxyeicosatetraenoic acids (HETEs) [30]. Among these, 20-HETE is the most extensively investigated and is known not only to influence vascular function but also to exert pro-inflammatory effects [46]. The epoxygenase activity of CYP enzymes—particularly members of the CYP2J and CYP2C families—converts AA into epoxides known as epoxyeicosatrienoic acids (EETs), including 5,6-, 8,9-, 11,12-, and 14,15-EET. These bioactive lipids are primarily generated in the liver, with physiologically significant levels also found in vascular tissue and in cardiomyocytes [47]. EETs are predominantly metabolized by soluble epoxide hydrolase (sEH), which converts them into their corresponding diols, the dihydroxyeicosatrienoic acids (DHETs) (Figure 2) [48]. Although AA-derived diols were once considered biologically weaker than their parent epoxides, it is now evident that EETs and their corresponding diols can even have opposing effects under certain conditions. Since EETs are known to promote vasodilation, the epoxygenase pathway and its metabolites have emerged as promising therapeutic targets for cardiovascular diseases (CVDs), including hypertension, heart failure, and stroke [49,50].

### 1.2. The Metabolic Pathways of Arachidonic Acid

#### 1.2.1. The COX Pathway

The term COX refers to the enzymes also known as prostaglandin G/H synthases (PGHS), which convert AA into the intermediates PGG_2_ and PGH_2_ [51]. These intermediates then serve as substrates for various downstream synthases that produce specific PGs, including PGE_2_, PGI_2_, PGD_2_, PGF_2_, and TXA_2_ (Figure 2) [52]. The primary distinction between the two COX isoforms is that COX-1 is broadly and constitutively expressed, whereas COX-2 is largely inducible [3,53,54], with certain notable exceptions [55,56]. Additionally, there are preferential interactions between COX enzymes and downstream synthases: COX-1 tends to couple more readily—but not exclusively—with thromboxane synthase, PGF synthase, and the cytosolic PGE synthase (cPGES) isoforms. In contrast, COX-2 more commonly channels PGG_2_/H_2_ toward prostacyclin synthase (PGIS) and the microsomal PGE synthase (mPGES) isoforms, both of which are frequently co-induced with COX-2 in response to cytokines and tumor promoters [57]. The overall pattern of prostanoid production is shaped by the varying expression of these enzymes in the cells present at sites of inflammation. Furthermore, the profile of prostanoid synthesis can change upon cellular activation. Another COX isoform, COX-3, a splice variant of COX-1 that also generates PGH_2_, has been identified. Its expression is reported to be higher in the microvessels of the brain and heart compared to larger conduit arteries [58]. Selective inhibition of the COX-2 enzyme, achieved through coxibs, such as celecoxib, can bypass the side effects of non-selective COX inhibitors, such as aspirin, which could lead to gastrointestinal erosions, and renal and hepatic insufficiency.

#### 1.2.2. The LOX Pathway

The arachidonate lipoxygenase (LOX) enzyme family includes several members, with arachidonate 5-lipoxygenase (5-LOX or ALOX5) being one of the most prominent. 5-LOX is a soluble, monomeric, non-heme iron-containing protein composed of 673 amino acids, with a molecular weight of approximately 78 kDa. It first catalyzes the oxygenation of arachidonic acid [AA; (5Z, 8Z, 11Z, 14Z)-eicosatetraenoate] to form 5-hydroperoxyeicosatetraenoate (5-HPETE) and subsequently converts this intermediate into 5,6-epoxyeicosatetraenoate (leukotriene A_4_, LTA_4_). These two reactions constitute the initial steps in leukotriene biosynthesis, producing potent mediators of inflammation [59,60].

Within this pathway, AA can be oxygenated at multiple positions. Specifically, an oxygen atom can be added to carbon atoms C-5, C-8, C-9, C-12, or C-15 by different LOX enzymes, which are named according to the carbon position they modify—for example, 5-LOX, 8-LOX, 9-LOX, etc. The site of oxygen insertion determines which of the four hydroperoxyeicosatetraenoic acids (HPETEs—5-, 8-, 12-, or 15-HPETE) is produced by the corresponding isoforms: 5-LOX, 8-LOX, 12-LOX, and 15-LOX. These HPETEs can subsequently be reduced by peroxidases to form monohydroxy eicosatetraenoic acids (HETEs) or further transformed into biologically active mediators, including LTs, lipoxins (LXs), hepoxilins and dihydroxyeicosatetraenoic acids (DHETEs). Specifically, 12-LOX and 15-LOX also generate LXs, oxo-ETEs, and DHETEs (Figure 2) [61].

Leukotrienes’ production depends on 5-LOX, which converts AA into 5-hydroperoxyeicosatetraenoic acid (5-HPETE). This intermediate is then transformed into leukotriene A_4_ (LTA_4_) by leukotriene synthase (LTS). LTA_4_ can be metabolized by two distinct enzymes: LTA_4_ hydrolase, which uses a water molecule to produce LTB_4_, a potent inflammatory mediator that acts through chemotaxis and degranulation of polymorphonuclear leukocytes (PMNs); and glutathione S-transferase, which conjugates glutathione to form LTC_4_. LTC_4_ can subsequently be further processed through the addition of amino acids to generate the peptidoleukotrienes LTD_4_ and LTE_4_ (Figure 2) [37,62]. 5-LOX inhibitors such as zileuton, are used instead of COX inhibitors, such as celecoxib, when the inflammation is driven by LTs, particularly in conditions like asthma, as opposed to inflammation primarily driven by PGs [63,64]. It is expected that via both COX-2 and 5-LOX inhibition the derived hybrid molecules will possess even more potent anti-inflammatory activities. Selective COX-2 inhibitors risk cardiovascular events (heart attack, stroke) but are gastrointestinal-sparing [65], while selective 5-LOX inhibitors have gastrointestinal, headache and pain side effects [66]. Known dual COX-2/5-LOX inhibitor licofelone tends to promote cardiovascular side effects, comparable to celecoxib [35]. Nonetheless, the potent anti-inflammatory activity they may promote is a major factor for developing such therapies.

#### 1.2.3. The CYP Pathway

CYP genes encode a large superfamily of mixed-function monooxygenases, encompassing over 6000 distinct enzymes [67]. The CYP pathway is best recognized for its involvement in metabolizing lipophilic xenobiotics—such as pharmaceuticals and chemical carcinogens—as well as a wide range of endogenous molecules, including steroids, fat-soluble vitamins, fatty acids, and biogenic amines [68,69]. The expression and enzymatic activity of CYPs are regulated by hormones, growth factors, and various transcription factors, and many CYP subfamilies exhibit intricate patterns of tissue-specific and developmental-stage–specific expression [70].

Cytochrome P450 (CYP) enzymes contain a heme-iron center and are expressed predominantly in the liver, as well as in several other tissues, where they play a key role in detoxifying harmful substances [71]. The CYP450 pathway involves two main enzymatic activities: cytochrome P450 epoxygenases [72] and P450 ω-hydroxylases (Figure 2) [73]. Epoxide metabolites are generated when CYP450 epoxygenases insert an oxygen atom into a carbon adjacent to one of the double bonds of AA. It generates four epoxyeicosatrienoic acid regioisomers—5,6-EET, 8,9-EET, 11,12-EET, and 14,15-EET—which act as autocrine and paracrine signaling molecules. In contrast, ω-hydroxylation by P450 ω-hydroxylases produces the hydroxyeicosatetraenoic acids 16-, 17-, 18-, 19-, and 20-HETE. EETs and HETE are produced in the kidneys [74]. EETs bind to PPAR_alpha_ [75,76] showcasing vasodilator effects. They can also bind to PPAR_Gamma_ [77] exhibiting anti-inflammatory and angiogenic functions, as well as inhibit Na^+^ transportation. On the other hand, HETE has vasoconstrictor effect [78].

### 1.3. COX-1 and COX-2 Enzyme Function

Cyclooxygenase enzymes exist in two forms, COX-1 and COX-2. COX-1 is expressed in nearly all tissues and generates PGs that support gastric mucosa regeneration and maintenance, regulate renal blood flow, and facilitate platelet aggregation [79].

COX-2 is an inducible enzyme expressed in inflamed and neoplastic tissues in response to stimuli such as epidermal growth factor (EGF), vascular endothelial growth factor (VEGF), fibroblast growth factor (FGF), and cytokines including tumor necrosis factor (TNF) and interleukins. Although several additional mechanisms for COX-2 regulation have been proposed, its mode of expression may differ between cell types [80].

NSAIDs like celecoxib and rofecoxib which are selective inhibitors of COX-2 do not possess the drawbacks of non-selective COX inhibitors such us indomethacin, sulindac, and piroxicam, as the stomach irritation and ulceration are side effects related to COX-1 inhibition [80,81]. That is why we will describe the current developments in the synthesis of dual inhibitors of the 5-LOX and only the COX-2 enzyme to achieve the anti-inflammatory effect desired.

### 1.4. COX-2 Active Site

#### Side Pocket

**Figure 3:**
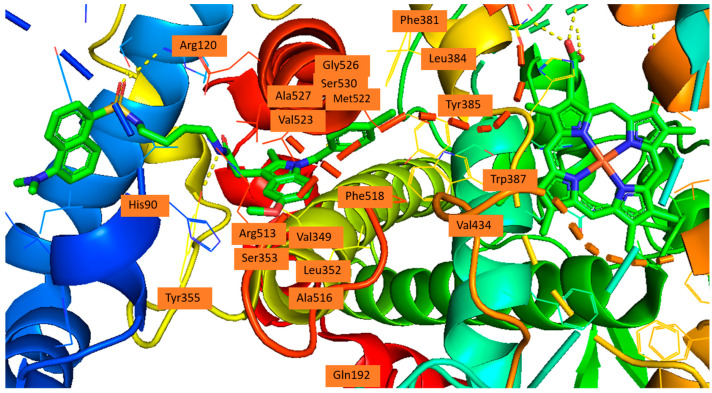
The active site of the Cyclooxygenase-2 (COX-2) enzyme bound with indomethacin-butyldiamine-dansyl conjugate. The active site’s major amino acid residues can be seen (Arg120, Val349, Leu352, Ser353, Tyr355, Phe381, Leu384, Tyr385, Trp387, Val434, Phe518, Met522, Val523, Gly526, Ala527 and Ser530) along with its side pocket (His90, Gln192, Leu352, Ser353, Tyr355, Arg513, Ala516, Phe518, and Val523) (PyMOL version 3.1, PDB; 6BL3).

The difference between the active sites of COX-1 and COX-2 is three residues near the active site: Ile434 (COX-1) versus Val434 (COX-2), Ile523 (COX-1) versus Val523 (COX-2), and His513 (COX-1) versus Arg513 (COX-2). These substitutions together create access to a side pocket above the constriction in COX-2 that is largely inaccessible in COX-1. This pocket, formed by residues His90, Gln192, Leu352, Ser353, Tyr355, Arg513, Ala516, Phe518, and Val523, is primarily targeted by the diarylheterocycle class of COX-2-selective inhibitors (Figure 3) [39,79,82,83].

### 1.5. 5-LOX Structure and Function

Arachidonate 5-lipoxygenase (5-LOX or ALOX5 or 5-LO) is a cytosolic protein at low intracellular calcium levels and translocates to the nuclear membrane when intracellular calcium concentrations are elevated [84]. Under oxidizing conditions, such as following diamide addition, ALOX5 forms covalent homodimers via disulfide bonds, resulting in markedly reduced catalytic activity. ALOX5 catalyzes the conversion of AA to the unstable hydroperoxyeicosatetraenoic acid (5-HPETE), which is rapidly transformed either into 5-HETE by glutathione peroxidase (GPX) [85] or into the 5,6-epoxide LTA_4_ by 5-LOX itself [59,85]. 5-LOX also participates in lipoxin biosynthesis by converting 15-HPETE into the epoxide 5(6)-epoxy-15-hydroxyeicosatetraenoic acid (5-epoxy-15-HPETE), which serves as one of the two precursors for the production of lipoxin A4 (LXA4) and lipoxin B4 (LXB4) [86].

Human 5-LOX (hALOX5, UniProtKB: P09917) is a monomeric, non-heme iron-containing enzyme. Six splice variant isoforms of ALOX5 have been reported; however, only isoform 1 encodes a fully functional protein [85]. Isoform 1 consists of 674 amino acids with a calculated molecular mass of approximately 78 kDa. To date, no X-ray crystal structures of human or other mammalian 5-LOX orthologs are available, likely due to the intrinsic instability of the enzyme [59]. Nevertheless, several stabilized 5-LOX structures have been resolved, including PDB entry 3O8Y where the destabilizing KKK_653–655_ sequence replaced with ENL, producing a species stable enough for crystallization [59].

### 1.6. Active Site of 5-LOX

**Figure 4:**
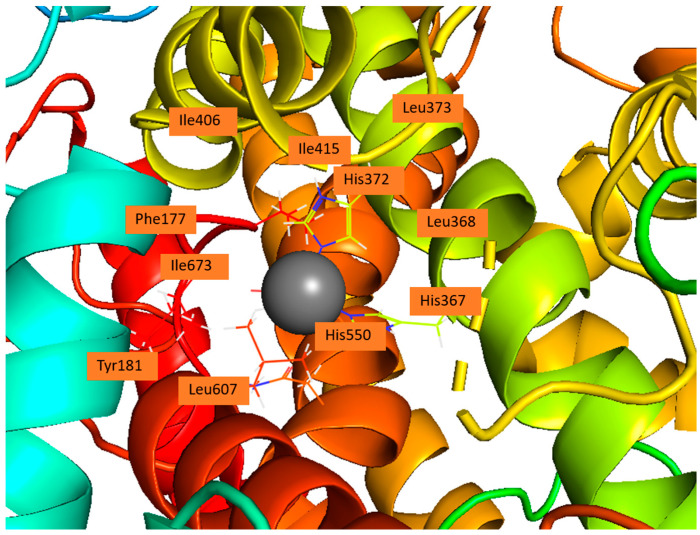
The active site of the 5-Lipoxygenase (5-LOX or ALOX5 or 5-LO) enzyme. The active site’s major amino acid residues can be seen (Phe177, Tyr181, His367, Leu368, His372, Leu373, Ile406, Ile414, His550, Leu607 and Ile673) (PyMOL version 3.1, PDB; 3O8Y).

The crystal structure reveals that the catalytic non-heme iron is coordinated by residues His367, His372, His550, and the carboxyl group of the C-terminal Ile673. Residues Leu368, Leu373, Ile414, Leu607, and Ile406 orient the substrate’s pentadiene moiety for catalysis. Additionally, Tyr181 and Phe177 act as a ‘cork’ at one end of the U-shaped substrate-binding cavity, helping to position the appropriate hydrogen atom for abstraction during the reaction (Figure 4) [59].

## 2. Novel Compounds Found to Be Selective and Potent Dual Inhibitors of the COX-2/5-LOX Enzymes from 2020–2024

A bibliographic search was carried out concerning all molecules that were synthesized from 2020 to 2024 and were potent dual inhibitors of the COX-2 and 5-LOX enzymes. Given the pro-inflammatory actions of LTs and prostanoids, agents capable of simultaneously inhibiting the production of both eicosanoid families (dual inhibitors) are expected to offer stronger anti-inflammatory effects while causing fewer adverse reactions than traditional NSAIDs [87]. The compounds studied in this review were selected based on their biological activity in vitro and/or in vivo experiments. Thus, compounds that present the lowest IC_50_ values were selected to be presented from each article. An interesting observation during the bibliographic search is that a large percentage of the literature focuses on plant extracts and characterization of these extracts for the reduction of inflammation and the inhibition of the COX-2 and 5-LOX enzymes. From all the articles which were reviewed, only those which exhibited the lowest IC_50_ values and were selective COX-2 inhibitors are going to be presented.

### 2.1. 2020

Sayed Jan et al. synthesized a novel series of thiazolidinone-based hybrids bearing a diarylpyrazole scaffold, aiming to develop dual inhibitors targeting both COX-2 and 5-LOX. Among the tested derivatives, compound **1** exhibited the most balanced dual inhibitory activity, with IC_50_ values of 0.98 ± 0.01 μM for COX-2 and 0.86 ± 0.01 μM for 5-LOX, as determined by in vitro enzyme assays. When compared with celecoxib (COX-2 IC_50_ = 0.28 ± 0.02 μM), compound **1** displayed a higher IC_50_ value, indicating lower COX-2 inhibitory potency (Table 1). Similarly, its 5-LOX IC_50_ value was greater than that of zileuton (IC_50_ = 0.63 ± 0.03 μM), demonstrating reduced potency toward the 5-LOX enzyme. Given these comparisons, further optimization is needed to improve the pharmacodynamic profile of compound **1**, particularly in enhancing its affinity for both targets. Molecular docking studies supported these results, revealing that **1** engaged in favorable binding within the COX-2 active site, through hydrogen bonds with His90, Gln192, Leu352, Ser353, and Arg513, while its interaction with the 5-LOX active site involved key residues such as Asn180, His550, Pro668, and Ala672. In vivo anti-inflammatory activity was assessed using the carrageenan-induced paw edema model, showing that **1** elicited 68.22% inhibition at the fifth hour at a dose of 100 mg/kg, compared to 57.64% for aspirin at the same time point. Additionally, **1** demonstrated anti-inflammatory activity in histamine-, bradykinin-, prostaglandin- and leukotriene-induced paw edema models, and exhibited antioxidant capacity in albumin denaturation and protease inhibition assays (IC_50_ = 16.89 μM and 15.29 μM, respectively). Overall, despite its moderate potency relative to reference inhibitors, compound **1** presents a balanced dual COX 2/5 LOX inhibitory profile and may benefit from structural refinement to enhance its therapeutic potential [88].

Gedawy et al. designed and synthesized a new series of pyrazole–thiazole hybrids aiming at dual inhibition of cyclooxygenase-2 (COX-2) and 5-lipoxygenase (5-LOX) enzymes. Among the tested molecules, compound **2** emerged as the most potent, exhibiting IC_50_ values of 0.01 ± 0.001 μM for COX-2 and 1.78 μM for 5-LOX in enzyme inhibition assays, in vitro (Table 1). Compared to the standard COX-2 inhibitor celecoxib (IC_50_ = 0.70 ± 0.03 μM), compound **2** displayed markedly superior potency, indicating a highly enhanced affinity for COX-2. In contrast, its 5-LOX inhibitory activity was weaker than that of licofelone (IC_50_ = 0.51 ± 0.03 μM), suggesting lower affinity for the lipoxygenase target. Therefore, while **2** is deemed worthy of in vivo and preclinical studies for its exceptional COX-2 activity, further optimization is needed to improve its pharmacodynamic profile toward 5-LOX inhibition. Molecular docking studies supported these findings, showing that **2** favorably occupied the COX-2 binding pocket, forming hydrogen bonds with residues such as Arg106, Tyr341, and Arg499, which are critical for COX-2 selectivity. In 5-LOX docking, ionic interaction with the catalytic Fe^2+^ and hydrogen bonding with Tyr181 and Asn425 were observed, though binding affinity was weaker compared to COX-2, in line with the experimental IC_50_ values. While in vivo anti-inflammatory activity was not reported exclusively for compound **2**, the strong in vitro potency and favorable binding profile of **2** position it as a highly selective COX-2 inhibitor with supplementary 5-LOX activity, representing a promising scaffold for dual-target anti-inflammatory drug development [89].

Sisa et al. developed a new series of benzoquinone and hydroquinone derivatives as potential dual inhibitors of COX-2 and 5-LOX. Among the tested compounds, compound **3** exhibited notable dual inhibitory activity, with IC_50_ values of 0.55 ± 0.19 μM for COX-2 and 0.28 ± 0.20 μM for 5-LOX, suggesting a balanced inhibition profile (Table 1). Compared to ibuprofen (COX-2 IC_50_ = 2.46 ± 0.97 μM), compound **3** demonstrated markedly greater potency, indicating enhanced COX-2 inhibitory activity. Similarly, its 5-LOX IC_50_ value was significantly lower than that of zileuton (IC_50_ = 0.66 ± 0.48 μM), reflecting superior inhibition of the lipoxygenase enzyme. Based on these findings, compound **3** is deemed worthy of further in vivo exploration and could serve as a promising candidate for clinical development as a dual COX-2/5-LOX inhibitor. Docking studies further confirmed the compound’s dual binding capability: within the COX-2 active site, **3** formed hydrogen bonds with Tyr355 and Arg120, while its hydrophobic tail occupied the substrate channel leading toward Tyr385, consistent with the known AA binding mode. In the 5-LOX binding pocket, compound **3** interacted with Tyr142 and formed aromatic contacts with Arg138, with the hydrophobic tail positioned in a lipophilic groove between the catalytic and membrane-binding domains. In vivo, compound **3** was evaluated in the carrageenan-induced paw edema model, where it produced 50.2% inhibition of edema at the third hour, compared to 58.7% for celecoxib and 52.8% for zileuton, indicating comparable efficacy. Altogether, compound **3** demonstrated promising characteristics as a dual COX-2/5-LOX inhibitor, supported by consistent in vitro, in vivo, and computational data [90].

Jacob et al. synthesized a novel series of 1,3,4-thiadiazole–pyrazole hybrids incorporating an arylsulfonamide moiety, aiming to develop dual COX-2 and 5-LOX inhibitors with anti-inflammatory activity. Among them, compound **4** displayed the most potent dual inhibition profile, with IC_50_ values of 0.07 ± 0.02 μM for COX-2, which was identical to that of the reference drug etoricoxib (IC_50_ = 0.07 ± 0.01 μM), and 0.29 ± 0.09 μM for 5-LOX, which was less potent than zileuton (IC_50_ = 0.15 ± 0.05 μM) (Table 1). Therefore, while the compound exhibits equally strong COX-2 inhibitory activity (same to etoricoxib), further optimization is needed to improve its pharmacodynamic profile with regards to the 5-LOX target. Molecular docking studies revealed hydrogen bond formation within the COX-2 active site between His356 and the nitro group of **4** (2.11 Å), as well as additional interactions with Gln192, Pro514, and Ser581, contributing to a binding energy of −8.87 kcal/mol. At the 5-LOX active site, binding was stabilized by hydrogen bonding with Trp147 and Arg411, as well as π–anion and van der Waals interactions involving the naphthoyl group, yielding a binding energy of −6.68 kcal/mol, which was better than zileuton (−6.43 kcal/mol). In vivo anti-inflammatory efficacy was confirmed using the carrageenan-induced paw edema model, where compound **4** showed 63% inhibition at a 20 mg/kg dose, demonstrating notable anti-inflammatory activity. The structure–activity relationship (SAR) analysis highlighted the critical role of the diphenylamino and arylsulfonamide groups in enhancing COX-2 selectivity and potency. Overall, compound **4** demonstrated highly potent COX-2 inhibition and moderate 5-LOX activity, with strong support from both experimental and computational studies, suggesting its potential as a lead scaffold for anti-inflammatory drug development [91].

Ahmad et al. synthesized a series of β-enaminone-based Michael adducts as potential dual inhibitors of COX-2 and 5-LOX, with anti-inflammatory and analgesic properties. Among them, compound **5** demonstrated significant dual enzyme inhibition, with IC_50_ values of 5.79 ± 0.23 μM for COX-2 and 1.06 ± 0.02 μM for 5-LOX, as determined via in vitro assays. Compared to indomethacin (COX-2 IC_50_ = 4.68 ± 1.08 μM), compound **5** exhibits slightly lower COX-2 inhibitory activity. For 5-LOX, its IC_50_ is higher than that of zileuton (IC_50_ = 0.69 ± 0.01 μM), suggesting moderate potency (Table 1). Therefore, further optimization is needed to improve its pharmacodynamic profile. In vivo analgesic activity was confirmed using acetic acid-induced writhing and formalin-induced paw licking tests, where compound **5** showed 48.71% inhibition at 50 mg/kg in the former and 33.66% (early phase) and 47.78% (late phase) inhibition in the latter, indicating central antinociceptive effects. Docking studies revealed stable binding within the active sites of both enzymes, involving hydrogen bonds and π-interactions with key residues, including Arg120 and Tyr355 in both COX isoforms, and Arg513 and Ser353 in COX-2. The compound also exhibited favorable drug-likeness and ADME properties in silico, including high predicted human intestinal absorption, blood–brain barrier permeability, and oral bioavailability. These findings suggest that compound **5** represents a promising scaffold that could benefit from structural refinement to enhance dual-target potency [92].

Qandeel et al. reported a new series of benzamide–thiazole hybrids as potential dual COX-2 and 5-LOX inhibitors. Within this series, compound **6** demonstrated selective COX-2 inhibition, exhibiting IC_50_ values of 5.45 ± 0.13 μM for COX-2 and 4.33 ± 0.08 μM for 5-LOX, indicating balanced and moderate dual inhibition (Table 1). When compared to celecoxib (COX-2 IC_50_ = 3.19 ± 0.06 μM), compound **6** was slightly less potent. Similarly, its 5-LOX inhibitory activity was lower than that of NDGA (IC_50_ = 2.46 ± 0.04 μM). Thus, further optimization is needed to improve its pharmacodynamic profile. Molecular docking studies supported these findings by revealing strong interactions of **6** with the active site of COX-2, particularly via hydrogen bonding with Lys83, Arg120, and Ser119, and similar stabilizing interactions within the 5-LOX pocket, including arene–cation and hydrogen bonding interactions with His372 and Gln557 near the catalytic iron center. SAR analysis emphasized that the nature and position of substituents on the benzamide ring played a critical role in modulating activity, with electron-withdrawing groups enhancing 5-LOX affinity. Although in vivo studies were not performed, the dual in vitro inhibitory activity and computational findings suggest that compound **6** represents a viable scaffold for further optimization as a dual COX-2/5-LOX anti-inflammatory agent [93].

Jacob et al. synthesized a novel series of thiazolo [3,2-a]pyrimidine–thiadiazole hybrids and assessed their potential as dual COX-2 and 5-LOX inhibitors. Among them, compound **7** showed the most promising inhibitory activity, with IC_50_ values of 0.09 ± 0.002 μM for COX-2 and 0.38 ± 0.01 μM for 5-LOX, indicating strong dual inhibition (Table 1). Compared to the reference drug etoricoxib (COX-2 IC_50_ = 0.07 ± 0.007 μM), compound **7** exhibited slightly lower COX-2 inhibitory potency. Similarly, its 5-LOX inhibitory activity was lower than that of zileuton (IC_50_ = 0.14 ± 0.01 μM). In addition to its in vitro profile, compound **7** demonstrated 60.82% edema inhibition in carrageenan-induced rat paw inflammation, confirming its in vivo anti-inflammatory efficacy. Molecular docking studies supported the biochemical findings, showing that **7** established hydrogen bonds with His351 in COX-2 and pi-sulfur interactions with Met145 in 5-LOX, along with hydrophobic interactions involving residues such as Tyr355, His90, and Ser581 in COX-2, and Trp147, Gln417, and Ala157 in 5-LOX. Structure–activity relationship (SAR) analysis revealed that the presence of electron-donating groups on the aryl moiety enhanced both potency and selectivity. Moreover, qRT-PCR analysis and reduction of PGE_2_ and LTB_4_ levels in treated tissues indicated that compound 7 significantly downregulated COX-2 and 5-LOX gene expression without affecting COX-1, supporting its selectivity. Although no clinical studies were reported, the potent dual inhibition, favorable docking profile, low acute toxicity, and improved gastric safety of compound **7** position it as a promising candidate for further development as a dual-action anti-inflammatory agent. Furthermore, compound **7** was shown to significantly downregulate pro-inflammatory cytokines including TNF-α, IL-1β, and IL-6 in carrageenan-induced inflamed paw tissue, supporting its anti-inflammatory action at the molecular level [94].

### 2.2. 2021

Sadiq et al. reported the design and biological evaluation of a series of succinimide-based compounds as dual COX-2/5-LOX inhibitors. Among these, compound **8** emerged as the most potent, exhibiting IC_50_ values of 0.051 ± 0.001 μM for COX-2 and 0.99 ± 0.10 μM for 5-LOX in enzyme inhibition assays, in vitro (Table 1). The COX-2 inhibitory activity of compound **8** was comparable to that of celecoxib (IC_50_ = 0.05 ± 0.01 μM), indicating potentially similar potency under comparable assay conditions. Its 5-LOX inhibition was slightly weaker than zileuton (IC_50_ = 0.64 ± 0.06 μM), suggesting good but lower affinity for the lipoxygenase target. Molecular docking studies confirmed strong binding affinities for both targets, with key interactions involving residues such as Arg120, His90, Gln192, and Leu352 in COX-2. The compound’s in vivo anti-inflammatory potential was validated through multiple rodent edema models. In the carrageenan-induced paw edema test, compound **8** demonstrated 57.13% inhibition at the third hour, closely approaching the activity of aspirin (up to ~53.64%). In models induced by prostaglandin E_2_ and leukotriene B_4_, it inhibited edema by 52.68% and 57.29%, respectively, although slightly lower than the reference drug montelukast (77.40%), the effect of compound **8** was still substantial. Additionally, moderate activity was observed in histamine—(23.55%) and bradykinin—(26.00%) induced edema, supporting its multi-pathway anti-inflammatory activity [95]. Given its comparable IC_50_ value to celecoxib for COX-2 and its moderate activity toward 5-LOX, compound **8** is a promising candidate for further preclinical development and investigation. These findings highlight compound **8** as a well-balanced dual inhibitor with robust in vitro and in vivo efficacy, making it a strong lead for anti-inflammatory drug development.

El-Miligy et al. synthesized a series of thymol–4-thiazolidinone hybrid conjugates and evaluated their biological activities. Among the tested derivatives, compound **9** exhibited the most promising dual inhibitory activity, with IC_50_ values of 0.091 ± 0.0016 μM for COX-2 and 3.54 ± 0.075 μM for 5-LOX, demonstrating clear COX-2 selectivity (Table 1). When compared with the reference COX-2 inhibitor celecoxib (IC_50_ = 0.045 μM), compound **9** displayed slightly weaker potency, indicating moderately reduced affinity toward the cyclooxygenase target. For 5-LOX, its IC_50_ value was higher than that of the reference inhibitor quercetin (IC_50_ = 3.34 ± 0.05 μM), suggesting lower inhibitory activity toward the lipoxygenase enzyme. Based on these comparisons, further optimization is needed to improve the pharmacodynamic profile of compound **9**. Molecular docking studies revealed high binding affinity for both targets, with docking scores of −8.09 kcal/mol (COX-2) and −6.93 kcal/mol (5-LOX), involving critical interactions with residues such as Leu338, Met508, Val509, Arg499 and Ser516 in COX-2 and Asn554, Gly174, Leu368, Val671, Gln413 in 5-LOX. The compound also demonstrated significant in vivo anti-inflammatory activity in the formalin-induced paw edema model, achieving 72.33% inhibition at the fourth hour, surpassing celecoxib (36.33%). Additionally, **9** displayed potent antinociceptive effects, reducing writhing responses by 74.5% in the acetic acid-induced writhing test. Overall, these results highlight compound **9** as a promising lead with validated in vitro, in vivo, and in silico activity, although structural refinement remains necessary to enhance its overall potency relative to standard inhibitors [96].

Bošković et al. designed a set of novel pyridazine-based derivatives bearing hydroxamic acid moieties to function as dual inhibitors of COX-2 and 5-LOX with additional iron-chelating properties. Among them, compound **10** displayed significant in vitro inhibitory activity, with IC_50_ values of 0.794 μM for COX-2 and 0.692 μM for 5-LOX, demonstrating balanced and potent dual inhibition (Table 1). The activity profile was found to be comparable to standard inhibitors celecoxib and zileuton, which were used as references. Molecular docking studies supported these findings, revealing a binding mode of compound **10** within the COX-2 side pocket, forming hydrogen bonds with His90, Arg513, Gln192, Ser353, and Leu352 and Phe518. In the case of 5-LOX, docking revealed metal-ligand coordination between the sulfohydroxamic group and the catalytic Fe^2+^ ion, along with hydrogen bonding to Ile673 and hydrophobic interactions with Leu368 and Leu414. The inclusion of sulfohydroxamic acid moiety was critical for metal ion chelation, a feature that not only contributed to 5-LOX binding affinity but also introduced potential antioxidant and anticancer properties. Although no in vivo studies were performed, the dual inhibitory potency, favorable docking profile, and metal-chelating ability suggest that compound **10** may serve as a promising scaffold for the development of multi-target anti-inflammatory agents [97].

Marques Duarte da Cruz et al. reported the design and synthesis of new pyrazole–thiazolidinone derivatives as dual COX-2/5-LOX inhibitors. Among the series, compound **11** exhibited promising dual enzymatic inhibition with IC_50_ values of 0.67 μM for COX-2 and 2.33 μM for 5-LOX, as measured by in vitro assays (Table 1). Although more potent than celecoxib (IC_50_ = 1.14 μM) and sodium meclofenamate (IC_50_ = 5.64 μM), compound **11** showed a favorable selectivity profile toward COX-2 and balanced activity overall. Molecular docking studies supported these findings, showing key hydrogen bonding and hydrophobic interactions within the COX-2 binding site and stable accommodation within the 5-LOX active site. The compound was also predicted to have favorable pharmacokinetic properties, including good oral bioavailability and low predicted toxicity, based on in silico ADME profiling. In vivo anti-inflammatory testing using the formalin-induced paw edema model showed that compound **11** was more effective than celecoxib and presented reduced gastric ulcerogenic effects. The dual inhibitory capacity, supported by both experimental and computational data, identifies compound **11** as a potential candidate for further development as an anti-inflammatory agent targeting both enzymatic pathways [98].

Francis et al. reported the isolation of Erectascalarane A (**12**), a marine-derived sesterterpenoid obtained from the sponge *Hyrtios erectus* and evaluated its anti-inflammatory potential through dual inhibition of COX-2 and 5-LOX. The compound exhibited moderate in vitro activity, with IC_50_ values of 800 ± 20 μM for COX-2 and 1210 ± 20 μM for 5-LOX, as determined by 2,7-dichlorofluorescein-based and spectrophotometric enzyme inhibition assays, respectively (Table 1). Compared to ibuprofen (COX-2 IC_50_ = 0.44 ± 0.01 mM), **12** is less potent, indicating that further optimization is needed to improve its pharmacodynamic profile. Also, against 5-LOX, its IC_50_ is significantly higher than that of ibuprofen (IC_50_ = 4.50 ± 0.05 mM, *p* < 0.05). Molecular docking simulations revealed strong binding affinity of compound **12** for COX-2, with a binding energy of −12.68 kcal/mol, inhibition constant (Ki) of 510.68 pM, and formation of four hydrogen bonds with key active site residues (Arg319, Trp125, Asp215). Despite its low potency, the marine origin and novel scalarane scaffold of **12** offer a unique chemotype for further exploration and optimization in anti-inflammatory drug discovery [99].

Mphahlele et al. synthesized a library of 1,3,4-oxadiazole derivatives and evaluated their potential as dual COX-2 and 5-LOX inhibitors. Among the tested molecules, compound **13** showed moderate dual activity, with IC_50_ values of 4.6 ± 1.45 μM for COX-2 and 15.0 μM for 5-LOX in vitro (Table 1). Compared to celecoxib (COX-2 IC_50_ = 0.62 ± 0.74 μM), **13** exhibits a significantly higher IC_50_, indicating lower potency. Similarly, its IC_50_ for 5-LOX is substantially greater than that of zileuton (IC_50_ = 0.42 ± 0.51 μM), suggesting poor inhibitory activity against this target. Therefore, further optimization is needed to improve its pharmacodynamic profile. A kinetic study revealed that **13** inhibits COX-2 competitively, with a Ki of 4.13 μM, as supported by Lineweaver–Burk and Dixon plots. Docking studies showed that **13** binds within the COX-2 active site (Val523, Arg513, and Val434) and engages hydrophobic interactions in the selectivity pocket. Additionally, halogen/hydrogen bonding interactions were also observed. For 5-LOX, molecular docking predicted coordination with residues near the catalytic center, though with less favorable binding energies (−8.15 kcal/mol), consistent with its weaker activity. While compound **13** does not match the potency of standard inhibitors, it provides a modest dual inhibition profile and represents a potential scaffold for further development [100].

Javed et al. designed and synthesized a library of pyrimidine–pyrrolidine hybrids as multifunctional anti-neuroinflammatory agents targeting both COX-2 and 5-LOX. Among them, compound **14** emerged as a highly potent dual inhibitor, exhibiting IC_50_ values of 0.029 ± 0.003 μM for COX-2 and 0.54 ± 0.0001 μM for 5-LOX, demonstrating superior selectivity for COX-2 over 5-LOX (Table 1). Compared to celecoxib (COX-2 IC_50_ = 50 ± 1 nM), compound **14** is more potent, while it also exhibits slightly better inhibitory activity than zileuton (5-LOX IC_50_ = 580 ± 14 nM). These findings suggest that the compound is deemed worthy for in vivo studies to take place so it can later get approval for clinical trials. Furthermore, molecular docking revealed that compound **14** established hydrogen bonds and π-π stacking interactions with critical residues within the COX-2 active site, including Arg120 and Gln192, while engaging hydrophobic and electrostatic contacts in the 5-LOX cavity. Overall, the extensive in vitro, in vivo, and in silico evaluations underscore compound **14** as a promising dual COX-2/5-LOX inhibitor with therapeutic potential in neuroinflammatory conditions [101].

### 2.3. 2022

Saraf et al. synthesized a series of 1,2,4-triazine-3-thiol derivatives incorporating benzenesulfonamide and thiazolidinone moieties as potential dual inhibitors of COX-2 and 5-LOX. Among them, compound **15** demonstrated potent dual enzymatic inhibition, with IC_50_ = 0.33 ± 0.02 μM for COX-2 and 4.90 ± 0.22 μM for 5-LOX, exhibiting greater selectivity toward COX-2 (Table 1). When compared with celecoxib (IC_50_ = 1.81 ± 0.13 μM), **15** displayed significantly lower IC_50_, indicating superior COX-2 inhibitory activity. Similarly, its 5-LOX IC_50_ value was markedly lower than that of zileuton (IC_50_ = 15.04 ± 0.18 μM), confirming enhanced dual inhibitory potency. Given its greater potency than both reference compounds, compound **15** is deemed worthy of further in vivo investigations and may serve as a viable candidate for preclinical development and potential progression toward clinical trials. Molecular docking studies showed that **15** binds favorably within the COX-2 active site, forming key hydrophobic interactions with Arg106, Ala513, Met508, Val335, Val509, Leu338, and Phe367, as well as a salt bridge with Arg499 and Tyr371, and a hydrogen bond with His75. For 5-LOX, interactions with His367, His372, Asn180, Asn554, Phe555, and Gln363 contributed to the observed affinity. In vivo evaluation using the carrageenan-induced paw edema model in rats revealed significant anti-inflammatory activity, with **15** producing 58.34% inhibition at the fifth hour. Additionally, **15** exhibited a high selectivity index (SI = 77.40), indicating favorable COX-2 selectivity over COX-1. Furthermore, molecular dynamics simulations over 100 ns confirmed the stability of **15** within both COX-2 and 5-LOX binding sites, with RMSD values remaining below 2.7 Å and consistent hydrophobic and hydrogen bond interactions, supporting the in silico reliability of the docking predictions. Overall, compound **15** presents a strong pharmacological profile as a selective and potent dual COX-2/5-LOX inhibitor, warranting further exploration as an anti-inflammatory drug candidate [102].

Bar et al. isolated a new series of β-carboline alkaloids from *Peganum harmala* seeds and evaluated their potential as dual COX-2/5-LOX inhibitors. Among these, compound **16** emerged as the most active, displaying IC_50_ values of 2.638 ± 0.07 μM for COX-2 and 1.63 ± 0.07 μM for 5-LOX in enzyme inhibition assays, in vitro (Table 1). When compared with celecoxib (COX-2 IC_50_ = 1.841 ± 0.05 μM) and zileuton (5-LOX IC_50_ = 0.71 ± 0.04 μM), compound **16** exhibited higher IC_50_ values for both enzymes, suggesting reduced inhibitory potency. Therefore, further optimization is needed to improve its pharmacodynamic profile, particularly to enhance affinity and efficacy against both COX-2 and 5-LOX. Molecular docking studies supported its binding affinity to both enzyme active sites, revealing arene-H and hydrogen bonding interactions with key residues including Ala527 in COX-2 and Ala672 and Phe610 in 5-LOX. The study also included SAR observations, indicating that the presence of electron-donating groups on the aromatic ring, especially the 7-OCH_3_ group, contributed positively to activity. Overall, compound **16** represents a promising scaffold for dual COX-2/5-LOX inhibition, warranting further development and in vivo evaluation [103].

Mahnashi et al. isolated a new class of phytosteroidal compounds from *Fragaria ananassa*, aiming to explore their potential as dual inhibitors of COX-2 and 5-LOX. Among them, compound **17** exhibited moderate in vitro activity, with IC_50_ values of 53 μM for COX-2 and 19 μM for 5-LOX, as determined by enzymatic inhibition assays (Table 1). In comparison to celecoxib (COX-2 IC_50_ ≈ 8.4 μM) and montelukast (5-LOX IC_50_ ≈ 7.7 μM), compound **17** showed significantly higher IC_50_ values, reflecting much lower inhibitory potency against both enzymes. Therefore, further optimization is needed to improve its pharmacodynamic profile. Docking studies suggested that compound **17** could interact with critical residues within both enzymes’ active sites. In COX-2, the compound engaged His90, Tyr355, Tyr385, Trp387, and Phe518 through π-alkyl interactions, while in 5-LOX, it interacted with Arg596 (hydrogen bond) and His372, His432 (π-alkyl interactions), albeit with lower binding affinity compared to known inhibitors. The lower experimental potency was consistent with the calculated binding scores and may be attributed to the rigidity of the steroidal scaffold or suboptimal orientation within the active sites. No in vivo studies were reported in this work. Despite its relatively weak activity, compound **17** provides a valuable scaffold for further structural modifications toward dual COX-2/5-LOX inhibition [104].

Javed et al. reported the design and synthesis of novel hybrid molecules derived from diclofenac, incorporating a pyrazoline–sulfonamide scaffold, aiming to achieve dual inhibition of cyclooxygenase-2 (COX-2) and 5-lipoxygenase (5-LOX). Among the synthesized compounds, compound **18** exhibited potent dual inhibitory activity, with IC_50_ values of 0.60 ± 0.03 μM for COX-2 and 0.98 ± 0.01 μM for 5-LOX in enzymatic assays, in vitro (Table 1). Compared to celecoxib (COX-2 IC_50_ = 0.05 ± 0.01 μM) and zileuton (5-LOX IC_50_ = 0.65 ± 0.04 μM), compound **18** demonstrated higher IC_50_ values for both enzymes, indicating lower inhibitory potency. Therefore, further optimization is needed to improve its pharmacodynamic profile. Molecular docking analysis demonstrated favorable binding of **18** within the COX-2 active site, as well as interaction with the iron center of 5-LOX, aligning with its observed experimental. SAR exploration indicated that the presence of an electron-withdrawing group on the aromatic sulfonamide moiety enhanced both potency and dual-target selectivity. Although the study did not include in vivo evaluation of anti-inflammatory efficacy, the strong in vitro profile, non-neurotoxicity in SH-SY5Y cells, and predicted BBB permeability, support further investigation of compound **18** as a potential anti-inflammatory lead with dual COX-2/5-LOX inhibition [105].

Alqahtani et al. reported the synthesis of a series of β-ketoester derivatives of N-aryl succinimides as potential anti-inflammatory agents via dual inhibition of COX-2 and 5-LOX. Among the evaluated compounds, compound **19** displayed relatively weak inhibitory activity, with IC_50_ values of 120 μM for COX-2 and 336 μM for 5-LOX (Table 1), making it significantly less potent than standard inhibitors celecoxib and zileuton. Compared to celecoxib (COX-2 IC_50_ ≈ 57.7 μM) and zileuton (5-LOX IC_50_ ≈ 81.3 μM), compound **19** exhibits approximately two- to four-fold higher IC_50_ values, indicating substantially reduced potency against both targets. However, in contrast to this weak in vitro potency profile, the compound demonstrated significant anti-inflammatory effects in vivo, particularly in carrageenan-induced paw edema and AA-induced ear edema models, with inhibition values comparable to aspirin and nimesulide, respectively. Despite the limited in vitro potency, molecular docking studies suggested potential interactions of compound **19** with active site residues of both enzymes. In COX-2, hydrogen bonding interactions were observed with Val523 and Ser353 and in 5-LOX the binding energy was −7.64 kcal/mol. These in silico results correlate well with the experimental observations and support the hypothesis of dual inhibitory behavior. No in vivo anti-inflammatory or pharmacokinetic assessments were reported. However, compound **19** also showed significant activity in mechanistic models using phlogistic agents like histamine, prostaglandin E2, and leukotriene, indicating multi-pathway anti-inflammatory potential. Given the high IC_50_ values and moderate to strong in vivo efficacy and docking profiles, compound **19** appears to have promise as a dual COX-2/5-LOX inhibitor scaffold [106].

Mahmood et al. synthesized a new series of pyridine–thiazole hybrids designed to act as dual COX-2 and 5-LOX inhibitors. Among these, compound **20** demonstrated the most potent dual inhibitory profile, with IC_50_ values of 0.18 ± 0.01 μM for COX-2 and 0.43 ± 0.02 μM for 5-LOX (Table 1). Compared to standard inhibitors, **20** was less potent than celecoxib (COX-2 IC_50_ = 0.042 ± 0.001 μM), indicating the need for further optimization of its pharmacodynamic profile. However, **20** was more potent than zileuton (5-LOX IC_50_ = 0.50 ± 0.02 μM), suggesting it is worthy of in vivo evaluation. Docking studies revealed that **20** interacted with the COX-2 active site via hydrogen bonding (Arg120, Tyr355, Val523) and hydrophobic interactions, while in 5-LOX it engaged in hydrogen bonding with Lys296 and His432, and π-π interactions with Trp599 and His432. Although no in vivo studies were included specifically for compound **20**, the compound also showed promising ADME profiles in silico. The combined enzymatic, structural, and docking data support **20** as a highly promising dual COX-2/5-LOX inhibitor with balanced activity and strong potential for further development [107].

Nagesh et al. reported the synthesis of a novel series of quinazoline-based Schiff base derivatives and evaluated their dual inhibitory activity against COX-2 and 5-LOX. Among them, compound **21** exhibited the most potent and balanced profile, with IC_50_ values of 39.43 ± 1.13 μM for COX-2 and 1.78 ± 0.09 μM for 5-LOX, indicating a higher selectivity for 5-LOX over COX-2 (Table 1). Compared to celecoxib (COX-2 IC_50_ = 35.56 ± 1.02 μM), compound **21** demonstrated similar inhibitory potency for COX-2. However, its 5-LOX inhibition was significantly weaker than that of indomethacin (IC_50_ = 0.51 ± 0.03 μM), although still within a pharmacologically relevant range, suggesting that further optimization is needed to improve its pharmacodynamic profile. Molecular docking studies supported these findings, showing that compound **21** established stable hydrogen bonds and hydrophobic interactions with key amino acids within the active sites of both enzymes. For COX-2, interactions were noted with His90, Tyr385, and Tyr355, while in 5-LOX, binding occurred in proximity to the iron center and catalytic residues such as Arg596, His432, His372, Phe359, and Trp599. The SAR analysis highlighted the importance of the presence of electron-donating methyl groups at the ortho positions of the phenyl ring and a fluoro group at the para position of the benzoyl ring in enhancing 5-LOX inhibition. Though in vivo studies were not conducted, compound **21** showed promising in vitro efficacy in both COX/5-LOX assays and exhibited favorable docking scores (–8.11 kcal/mol with 5-LOX), supporting its potential as a dual anti-inflammatory agent worthy of further pharmacological exploration [108].

### 2.4. 2023

Bošković et al. designed and synthesized a series of N-hydroxyurea derivatives, 3,5-di-tert-butylphenol derivatives and “type B hydroxamic acids” to evaluate their enzyme inhibition potential and redox properties against COX-2 and 5-LOX as anti-inflammatory drugs. Thirteen compounds were designed based on key structural features needed for dual COX-2 and 5-LOX inhibition, as well as antioxidant activity, and were subsequently synthesized and structurally characterized. Compounds (**22**, **23**, **24**, **25**, **26**, **27** and **28**) proved to be dual COX-2 and 5-LOX inhibitors. These compounds expressed good COX-2/COX-1 selectivity. Moreover, dual inhibitors **22**, **24**, **25**, **27** and **28** showed good antioxidant properties. Specifically, compounds **24** (IC_50_ COX-2; 5.26 ± 0.34 μM and 5-LOX; 1.73 ± 0.64 μM) and **23** (IC_50_ COX-2; 6.72 ± 0.79 μM and 5-LOX; 1.62 ± 0.67 μM) (Table 1) showed the best inhibitory activity of COX-2 and 5-LOX enzymes within the synthesized group of compounds. However, they do not exhibit the potency which reference compounds celecoxib (COX-2; IC_50_ = 0.07 ± 0.01 μM) and zileuton (5-LOX; IC_50_ = 0.36 ± 0.10 μM) showcase. Furthermore, their redox activity was evaluated. The synthesized compounds could be ranked according to their antioxidant potential (oxy score, OS, median values) in the following order: **27** > **28** > **24** > **26** [109]. Nontheless, further optimization of these derivatives could manifest even better anti-inflammatory properties in the long run.

A series of novel indole and indazole arylamide benzoic acid analogues were designed and synthesized by Du et al. as dual COX-2/5-LOX inhibitors and were evaluated for their anti-inflammatory properties in vivo. Compounds **29** and **30** demonstrated notable anti-inflammatory effects in a xylene-induced mouse auricular edema model. Both molecules also showed moderate COX-2 inhibition in vitro (IC_50_ = 0.54  ±  0.033 μM and 0.32  ±  0.039 μM, respectively), although less potent than celecoxib (IC_50_ = 0.010  ±  0.0005 μM). Among them, **30** displayed greater COX-2 selectivity (COX-1/COX-2 selectivity index = 7.89) along with moderate 5-LOX inhibition (IC_50_ = 0.22 ± 0.021 μM) (Table 1). In comparison to zileuton (IC_50_ = 0.036 ± 0.002 μM), compound **29** emerged as the strongest 5-LOX inhibitor within the series (IC_50_ = 0.077  ±  0.0015 μM), but still not as strong as zileuton [110]. Further structural optimization is needed.

A new series of thymol–1,5-disubstituted pyrazole hybrids was developed by El-Miligy et al., as dual COX-2/5-LOX inhibitors. Compounds **31**, **32**, **33** and **34**, exhibited potent COX-2 inhibition in vitro (IC_50_ = 0.068  ±  0.01, 0.043  ±  0.001, 0.063  ±  0.001, and 0.045  ±  0.01 µM, respectively), with activities comparable to celecoxib (IC_50_ = 0.045  ±  0.007 µM). Their selectivity indices (151.5, 316.5, 204, and 268.8) were also similar to that of celecoxib (IC_50_ = 0.045 ± 0.007 µM) (327). All synthesized compounds demonstrated stronger in vitro 5-LOX inhibition (**31**; IC_50_ = 3.05  ±  0.067 µM, **32**; IC_50_ = 1.58  ±  0.026 µM, **33**; IC_50_ = 1.91  ±  0.053 µM and **34**; IC_50_ = 1.60  ±  0.042 µM) than the reference compound quercetin (3.34  ±  0.05 µM) (Table 1). Moreover, they showed enhanced in vivo suppression of formalin-induced paw edema compared with celecoxib. Compounds **31**, **32**, and **34** additionally displayed an improved gastrointestinal safety profile, causing no ulceration in fasted rats, similar to celecoxib and diclofenac sodium [111].

Badawi et al. reported new pyridazine-based sulphonamides as potential multi-target anti-inflammatory candidates. Specifically, compounds **35** (IC_50_ COX-2; 0.05 μM and 5-LOX; 3 μM) and **36** (IC_50_ COX-2; 0.06 μM and 5-LOX; 2.5 μM) (Table 1) showed the same inhibitory activity for COX-2 enzyme as celecoxib (IC_50_ = 0.05 μM) and greater inhibitory activity than 5-LOX zileuton (IC_50_ = 3.5 μM). Further in vivo investigations for pyridazine sulphonates **35** and **36** revealed their ability to reduce the total number of writhing in mice, the rat paw edema, and the serum levels of the inflammatory mediators (TNF-α and IL-1β), which highlights their analgesic and anti-inflammatory activities [112]. Thus, these compounds are deemed worthy for further in vivo and clinical studies.

Ragab et al. presented the design and synthesis of a novel series of 4-(5-amino-pyrazol-1-yl)benzenesulfonamide derivatives with COX-2, 5-LOX and carbonic anhydrase (CA) inhibitory activities as potential multi-target anti-inflammatory agents. All synthesized pyrazole derivatives were tested for their inhibitory effects on COX-1, COX-2, and 5-LOX. Among them, compounds **37**, **38** and **39** demonstrated the strongest COX-2 inhibition, with IC_50_ values of 0.049 ± 0.001, 0.060 ± 0.002, and 0.060 ± 0.001 μM, respectively. These compounds also showed potent 5-LOX inhibition (IC_50_ = 2.4 ± 0.1, 1.9 ± 0.1, and 2.5 ± 0.1 μM, respectively) and exhibited excellent COX-2 selectivity, with selectivity indices (COX-1/COX-2) of 212.24, 208.33 and 158.33 (Table 1). All of these molecules were slightly less potent for COX-2 inhibition when compared with reference compound celecoxib (IC_50_ = 0.045 ± 0.001 μM), but more potent than reference compound zileuton (IC_50_ = 3.5 ± 0.2 μM) for 5-LOX inhibition. The serum level of the inflammatory mediators tumor necrosis factor-alpha (TNF-α) and interleukin one beta (IL-1β) was, also, measured. Pyrazoles **37** and **38** significantly reduced the inflammatory mediators which confirms the anti-inflammatory activities of these compounds [113].

A new set of N-(benzenesulfonyl)acetamide derivatives was designed and synthesized by Chen et al. Among them, compounds **40** and **43** demonstrated strong inhibitory effects on COX-2, 5-LOX, and TRPV1. Compounds **40** (IC_50_ COX-2; 0.011 μM and 5-LOX; 0.46 μM), **41** (IC_50_ COX-2; 0.023 μM and 5-LOX; 0.31 μM), **42** (IC_50_ COX-2; 0.076 μM and 5-LOX; 0.12 μM), **43** (IC_50_ COX-2; 0.025 μM and 5-LOX; 0.52 μM) showcased a strong affinity for the COX-2 and 5-LOX enzyme (Table 1). However, only compound **40** showed higher inhibitory potency to celecoxib (COX-2; IC_50_ = 0.013 μM), while only compounds **41** and **42** were more potent inhibitors of 5-LOX when compared to zileuton (5-LOX; IC_50_ = 0.39 μM). Moreover, **40** exhibited superior bioavailability along with potent anti-inflammatory analgesic activity in vivo. Specifically, **40** was capable of ameliorating formalin-induced pain and inhibiting capsaicin-induced ear edema [114]. All of the aforementioned compounds are strong inhibitors of COX-2 and 5-LOX and further in vivo and toxicology studies could lead them to the clinical phase of research.

Novel series of N-methylsulfonylindole derivatives were synthesized by Philoppes et al. Compounds **44** (IC_50_ COX-2; 0.819  ±  0.04 μM and 5-LOX; 4.08  ±  0.22 μM) and **45** (IC_50_ COX-2; 0.67  ±  0.04 μM and 5-LOX; 1.10  ±  0.06 μM) showed the best affinity for the COX-2 enzyme amongst all studied compounds (Table 1), although they were not as potent as the reference compound celecoxib (IC_50_ = 0.46  ±  0.02 μM). At the same time none of the studied compounds showed higher potency than zileuton (IC_50_ = 0.58  ±  0.03 μM) for the 5-LOX enzyme and further optimization is needed. The in vitro antioxidant activity of the compounds was determined by measuring the reduction capacity of DPPH radicals spectrophotometrically at 540 nm. Trolox, a water-soluble analogue of vitamin E, was used as the reference standard. Compound **44** showcased moderate anti-oxidant activity when compared to the rest compounds, while the anti-oxidant activity of compound **45** was not able to be detected, since it was DMSO and methanol insoluble. Metabolic liability was assessed through Phase I metabolism predictions. Most compounds were predicted to inhibit the cytochrome P450 isoform CYP2C9, whereas none showed inhibitory potential toward other major isoforms, including CYP2C19, CYP2D6, and CYP3A4 [115].

Ten new diarylthiophene-2-carbohydrazide derivatives were synthesized and evaluated by Coskun et al. for their pharmacological activity. Compounds **46** (IC_50_ COX-2; 10.13 ± 0.25 μM and 5-LOX; 2.60 ± 0.11 μM) and **47** (IC_50_ COX-2; 13.86 ± 0.76 μM and 5-LOX; 3.30 ± 0.07 μM) showed the best affinity for the COX-2 enzyme amongst all studied compounds, although they were 30-fold less potent than the reference compound celecoxib (IC_50_ = 0.07 ± 0.01 μM). At the same time none of the studied compounds showed higher potency than zileuton (IC_50_ = 0.36 ± 0.10 μM) for the 5-LOX enzyme [116]. Thus, further optimization is of the utmost importance in order for these molecules to attain a better pharmacodynamics profile.

Soliman et al. utilized *Rhizopus oryzae* to biotransform carotol, the major sesquiterpenoid of carrot fruit essential oil. This procedure yielded two new lactate derivatives, one of which was 9α-hydroxydaucol-9-lactate (compound **48**). All compounds showed considerable in vitro inhibition of COX-2, 5-LOX, and butyrylcholinesterase (BChE). These targets were studied as cholinergic deficiency and neuroinflammation are key detrimental processes involved in the etiology of Alzheimer’s disease (AD). Compound **48** (IC_50_ COX-2; 9.03 ± 1.196 μM and 5-LOX; 0.56 ± 1.074 μM) showcased the most potent inhibitory activity for COX-2 amongst all studied compounds, but it still fell short in activity when compared to celecoxib (IC_50_ COX-2; 1.61 ± 0.052 μM) by 9-fold (Table 1). On the other hand, its potency for the inhibition of 5-LOX was comparable to diclofenac (IC_50_ 5-LOX; 0.42 ± 0.017 μM) [117]. This study is a good indication that natural products and natural product derivatives can constitute effective treatments for various diseases. In this case, carrot essential oil and its carotol-derived compounds may serve as potential therapeutic agents for neurodegenerative diseases linked to neuroinflammation.

A fragment-merging strategy was employed by Elgohary et al. to design thiazole/thiazolidinone–pyrazoline hybrids as dual COX-2/5-LOX inhibitors. Among them, compounds **49** (IC_50_ COX-2; 0.06 ± 0.004 μM and 5-LOX; 4.36 ± 0.37 μM), **51** (IC_50_ COX-2; 0.04 ± 0.004 μM and 5-LOX; 4.86 ± 0.32 μM), **52** (IC_50_ COX-2; 0.03 ± 0.003 μM and 5-LOX; 4.86 ± 0.25 μM) and **54** (IC_50_ COX-2; 0.06 ± 0.003 μM and 5-LOX; 3.54 ± 0.35 μM) (Table 1) showed the strongest COX-2 inhibition (IC_50_ = 0.03–0.06 μM) with high selectivity and potent 5-LOX inhibition when compared to reference compounds celecoxib (IC_50_ COX-2; 0.04 ± 0.003 μM), indomethacin (IC_50_ COX-2; 0.06 ± 0.005 μM) and quercetin (IC_50_ 5-LOX; 3.81 ± 0.26 μM). Other compounds like **50** and **53** were also potent inhibitors of the aforementioned enzymes. In vivo pharmacological evaluation revealed that compound **51** exhibited superior anti-inflammatory efficacy compared to the standard drugs indomethacin and celecoxib. Moreover, **51** demonstrated an improved safety profile, as evidenced by histopathological examination of the rat stomach, liver, and kidney, along with assessments of liver and renal function markers (AST, ALT, and creatinine) and cytotoxicity studies. All these results indicate that compound **51** can further be optimized and possibly used in clinical studies in the future [118].

New derivatives of the antitubercular and anti-inflammatory drug, 4-aminosaliclic acids (4-ASA) were synthesized, characterized, and evaluated for these activities by the research group of Qahtan et al. In vivo and in vitro evaluations of anti-inflammatory activity demonstrated that compounds **55** (IC_50_ COX-2; 1.16 ± 0.05 μM and 5-LOX; 4.38 ± 0.2 μM), and **56** (IC_50_ COX-2; 4.54 ± 0.21 μM and 5-LOX; 9.64 ± 0.45 μM) were the most active (Table 1), exhibiting potent cyclooxygenase-2 (COX-2) and 5-lipoxygenase (5-LOX) inhibition when compared to celecoxib (IC_50_ COX-2; 1.16 ± 0.05 μM) and zileuton (IC_50_ 5-LOX; 0.62 ± 0.03 μM). **56** displayed low cytotoxicity against normal cells, providing a high therapeutic index. These results indicate that compounds **55** and **56** are potential lead compounds for the discovery of dual anti-inflammatory and antitubercular drug candidates [119].

From the dichloromethane (DCM) fraction of the crude ethanolic extract of *Caralluma awdeliana*, four pregnane glycosides and one flavone glycoside were isolated by El-Shiekh et al. using a bio-guided isolation approach. The various extracts of *C. awdeliana* were then evaluated through in vitro enzyme inhibition assays targeting cholinesterases (AChE and BChE) as well as key inflammatory enzymes (COXs and 5-LOX). From these studies, compound **57** (IC_50_ COX-2; 1.07 ± 0.01 μM and 5-LOX; 26.30 ± 1.79 μM) (Table 1) showcased an inhibitory activity very closely potent to that of celecoxib (IC_50_ COX-2; 0.78 ± 0.02 μM) and more potent than zileuton (IC_50_ 5-LOX; 169.28 ± 2.11 μM) [120]. This is yet another example of a natural product extract which showcases better inhibitory activity than that of known dugs.

### 2.5. 2024

Al-Wahaibi et al. synthesized and investigated 15 diaryl-1,2,4-triazolo [3,4-a]pyrimidine hybrids in vitro as selective COX-2/sEH/5-LOX inhibitors. From their studies, compounds **58** (IC_50_ COX-2; 15.20 μM and 5-LOX; 3.50 ± 0.04 μM), **59** (IC_50_ COX-2; 11.60 μM and 5-LOX; 3.05 ± 0.03 μM) and **60** (IC_50_ COX-2; 10.50 μM and 5-LOX; 2.90 ± 0.03 μM) were the most active, exhibiting potent COX-2 and 5-LOX inhibition (Table 1) when compared to celecoxib (IC_50_ COX-2; 42 μM) and quercetin (IC_50_ 5-LOX; 3.35 ± 0.05 μM). In vivo investigations also demonstrated that these compounds were the most efficacious as analgesic/anti-inflammatory derivatives with a high cardioprotective profile against cardiac biomarkers and inflammatory cytokines. Thus, these compounds are good candidates for developing anti-inflammatory agents with less gastrointestinal and cardiovascular side effects [121].

Quinone-containing compounds were synthesized and evaluated by Chaaban et al. in order to investigate the anti-inflammatory activity of this important scaffold. Compounds **61** (IC_50_ COX-2; 0.041 μM and 5-LOX; 1.96 μM), **62** (IC_50_ COX-2; 0.042 μM and 5-LOX; 1.59 μM), **63** (IC_50_ COX-2; 0.041 μM and 5-LOX; 1.74 μM), **64** (IC_50_ COX-2; 0.044 μM and 5-LOX; 2.66 μM) and **65** (IC_50_ COX-2; 0.078 μM and 5-LOX; 3.11 μM), exhibited greater inhibitory potency for the COX-2 and 5-LOX enzymes (Table 1) when compared to celecoxib (IC_50_ COX-2; 0.045 μM) and zileuton (IC_50_ 5-LOX; 3.50 μM). The obtained in vivo paw edema results showed high inhibitory percentage for the compounds **63** and **64** with the significant lower TNF-α relative mRNA expression for compounds **63** and **64** making them great lead compounds for further evaluation [122].

New thymol–3,4-disubstitutedthiazole hybrids were synthesized as dual COX-2/5-LOX inhibitors, by El-Miligy et al. Compounds **66** (IC_50_ COX-2; 0.037  ±  0.007 μM and 5-LOX; 2.51  ±  0.015 μM), **67** (IC_50_ COX-2; 0.042  ±  0.001 μM and 5-LOX; 2.47  ±  0.06 μM), **68** (IC_50_ COX-2; 0.046  ±  0.0016 μM and 5-LOX; 1.75  ±  0.06 μM) and **69** (IC_50_ COX-2; 0.039  ±  0.001 μM and 5-LOX; 1.53  ±  0.02 μM), exhibited greater inhibitory activity for the COX-2 and 5-LOX enzymes (Table 1) when compared to celecoxib (IC_50_ COX-2; 0.045  ±  0.001 μM) and quercetin (IC_50_ 5-LOX; 3.34  ±  0.05 μM). All these compounds also possessed in vivo inhibition of formalin-induced paw edema higher than celecoxib. Moreover, compounds **66** and **69** showed the same gastrointestinal safety profile as celecoxib and diclofenac sodium in a population of fasted rats [123]. Thus, these molecules should proceed to further in vivo and clinical testing.

Khadri et al. synthesized and examined thiophene bearing pyrazole derivatives for their in vitro cyclooxygenase, 5-lipoxygenase, and tumor inducing factor-α inhibitory activities followed by the in vivo analgesic, anti-inflammatory, and ulcerogenic evaluations. Compounds **70** (IC_50_ COX-2; 1.76  ±  0.03 μM and 5-LOX; 0.27  ±  0.06 μM) and **71** (IC_50_ COX-2; 1.01  ±  0.02 μM and 5-LOX; 0.29  ±  0.06 μM) (Table 1), exhibited the greatest potency among all synthesized molecules, but it had lower inhibitory activity for the COX-2 enzyme when compared to reference compound celecoxib (IC_50_ COX-2; 0.039  ±  0.01 μM), but greater affinity for 5-LOX when compared to nordihydroguaiaretic acid (NDGA) (IC_50_ 5-LOX; 0.49  ±  0.22 μM) [124]. Consequently, these molecules need to undergo further optimization so they can have a better pharmacodynamic profile.

Mahnashi et al. synthesized seven new thiazole derivatives for the development of novel anti-inflammatory drugs. Compounds **72**, and **73** were dominant and selective COX-2 inhibitors with the lowest IC_50_ values (**72**; 0.83 ± 0.03 μM and **73**; 0.76 ± 0.17 μM) and selectivity index (SI) values of 112, and 124, respectively (Table 1). In the 5-LOX results, once again, compounds **72** and **73** were dominant with IC_50_ values of 23.08 and 38.46 μM, respectively. Regarding the affinity for the COX-2 and 5-LOX enzymes, in both cases **72** and **73** showcased a lot higher IC_50_ values compared to celecoxib (0.05 ± 0.01 μM) and zileuton (11.00 ± 0.12 μM). Thus, these compounds are in need of further optimization. Nonetheless, compounds **72** and **73** were significant anti-inflammatory anti-nociceptive agents in animal models [125].

Akbar et al. synthesized and evaluated amide-based carboxylate derivatives for their anti-inflammatory potentials using in vitro, in vivo and in silico studies. Compound **74** (IC_50_ COX-2; 0.288 μM and 5-LOX; 0.83 μM) exhibited lower inhibitory activity for the COX-2 enzyme (Table 1) when compared to reference compound celecoxib (IC_50_ COX-2; 0.041 μM), but greater affinity for 5-LOX when compared to montelukast (IC_50_ 5-LOX; 0.194 μM). All the synthesized compounds were screened for their in-vivo anti-inflammatory potential and from this it was deduced that the compounds were excellent anti-inflammatory agents in carrageenan induced paw edema at various doses [126].

Table 1 summarizes the most potent dual inhibitors reported between 2020–2024, including their compound codes, IC_50_ values (expressed in μM), and corresponding references.

## 3. Discussion

### 3.1. Structural Observations

Almost all studied molecules have showcased excellent inhibitory activity against COX-2 and 5-LOX enzymes. Compounds **12**, **17**, **19** and **21** had the least affinity for both of these enzymes, while compounds **13**, **22**, **25**, **26**, **46**–**48** showed mediocre affinity for the targets. Compound **26** should be considered as a very low affinity inhibitor, regarding its 5-LOX inhibition. Compounds **27** and **28** showed very low inhibitory activity for the COX-2 enzyme and compounds **57**, **72** and **73** had very low affinities for the 5-LOX enzyme. Compounds **5**, **6**, **23**, **24** and **46**–**48** showed mediocre inhibitory activity for the COX-2 enzyme. The rest of molecules studied in this review are of excellent inhibitory activity towards both enzymes (COX-2 and 5-LOX).

Closer inspection of the chemical structures of the molecules with the highest affinity for the COX-2 and 5-LOX enzymes reveals some interesting features of these compounds. Compounds **2**, **7**, **8**, **14**, **36**–**43**, **54**, **61**–**69** have four common and repeatable characteristics between them. Thus, we decided to name these observations: “*The Rule of Four for Inflammation*”. (1) All of these molecules have a relatively extended carbon backbone, spanning about 18 carbon atoms. This enables them to penetrate deep into the enzyme cavity and interact with a greater number of amino acids. Above a certain length, however, this property is lost and the result is a high IC_50_ value, as can be observed with molecule **21** (COX-2 inhibition) and **57** (5-LOX inhibition). (2) Another factor that leads to improved inhibition of the studied enzymes, is that the compounds reviewed have approximately 6 bonds in their backbone that are amenable to rotation, thus increasing the degrees of freedom of the molecules. This provides further possibilities for interactions within the active cavity, in contrast to when the structures are compact (see high IC_50_ values in the cases of compounds **12** and **17**). (3) Furthermore, all of these compounds constitute relatively extensive aromatic systems, while in several positions they carry aromatic rings and between them, a single or double bond. (4) Finally, the characteristic that can be observed that is perhaps what differentiates a low IC_50_ value from a very low one, is the existence at one end of the molecule of a benzenesulfonamide group, or instead of an amide, acid, etc. group, while it is directly linked via a single bond to a five-membered heterocyclic ring (pyrazole-like) which is substituted at its 5-position (carries either an amino group, or methyl group, etc.). Connection between the two groups (benzenesulfonamide-group and pyrazole-ring) can occur through the 1-position of the pyrazole-like ring and the 4-position of the benzenesulfonamide group.

Based on the data collected and the aforementioned analysis, the compounds possessing the lowest IC_50_ values are compounds **2** and **40** (IC_50_ approximately 0.01 μM).

### 3.2. Computational Proofs

In order to realize our observations, and prove the validity of “*The Rule of Four for Inflammation*”, we performed computational docking simulations utilizing Maestro software version 2021-02 from Schrödinger [127]. Specifically, we docked the compounds with the highest and lowest affinities for the COX-2 and 5-LOX receptors, so that we can observe their binding capacities. The compounds were separated into classes and groups. The class with the highest affinities for these enzymes (according to their IC_50_ values), was subdivided into 2 separate groups, according to their agreement to the proposed rules. The same procedure was followed with the class for the lowest affinities towards these receptors, leading to the creation of 4 groups.

From the results of the in silico docking simulations, the binding energies of the compound-target complexes were inserted in Table 2. The binding energy values are in agreement with the experimentally observed IC_50_ values. Some interesting observations can be made. For groups A and B both IC_50_ values and docking energies are in agreement within each group, with only minor differentiations. Specifically, while compound **2** has a lower IC_50_ value when compared to **41** and **69** for COX-2, the binding energies have the exactly opposite order. The difference is not big, since it is only 1 kcal/mol difference, but still exists. Nonetheless, both IC_50_ and binding scores agree on the potency of these molecules for the COX-2 active site. Furthermore, the placement of the sulfonamide group in the middle of the compound’s structure, in some cases increases the inhibitory potency of the structure for the studied enzymes (see compound **40**), while in others the positioning of the sulfonamide group at the end of the structure increases it (see compound **41** within Group A). Positioning of the sulfonamide group in the middle of the structure is perhaps best, in order to attain more potent dual inhibitors, as can be observed from the IC_50_ values for 5-LOX of compounds **2** and **41**.

The compounds with the lowest affinity for COX-2/5-LOX showcase a very similar trend, when examined. When compound **10** (Group A’) is compared to molecules **16** and **60**, the effect of the five-membered heterocyclic ring (pyrazole-like) becomes evident. From the IC_50_ values it seems that the existence of the five-membered heterocyclic ring upgrades the affinity of the compound for the receptor. The same can be said for the existence of the sulfonamide group in compound **10** when compared to **16** and **60**, but to a lesser extent when compared to the role and effect of the ring. In compound **60**, a pyrazole- kind of ring does exist, but it is not able to rotate. Thus, in order for the five-membered ring to exert its potency, it needs to have certain degrees of freedom (one single bond connected between the rest of the compound and the ring).

The only instance, where the computational result is not in agreement to the experimental one is for compound **21** (Group D’). This is a false positive result (binding energy for COX-2; −12.526 kcal/mol), since the larger a chemical structure is and the more chemical groups it contains, the greater its interactions with the active site of a target-biomolecule will be in silico. The case of compound **21** also shows that even if three of the four rules are obeyed, if the first rule is not followed then this will result in low affinity for the target. In fact, excessive molecule elongation leads to even worse interaction with the active site of the COX-2/5-LOX enzymes than when the molecules are smaller than the desired size (around 18 carbon atoms in length).

**Figure 5:**
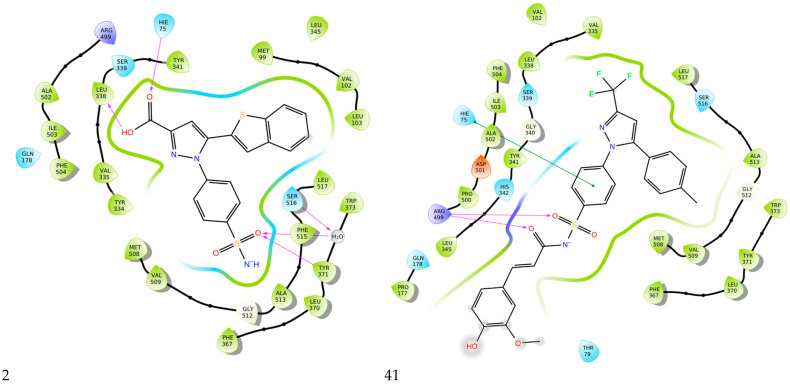

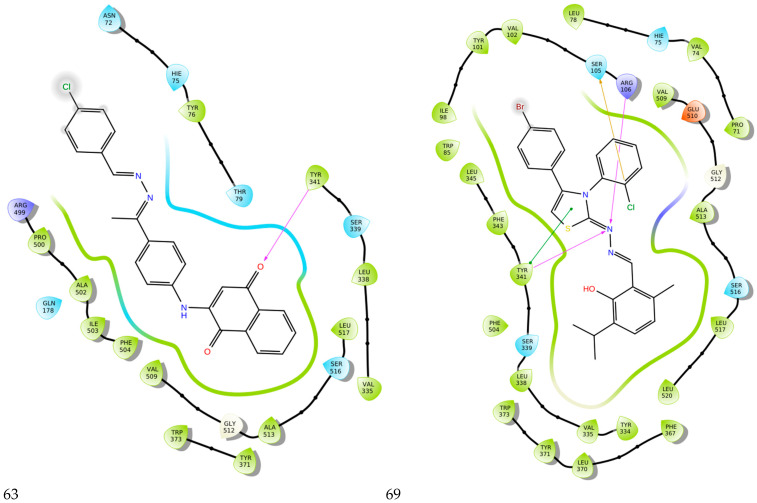
2D ligand interactions’ diagrams of studied compounds with the highest affinity for the COX-2 enzyme (PDB ID: 3LN1). It can be observed that all these compounds (**2**, **41**, **63** and **69**) are able to penetrate deeply into the active site cavity of the COX-2 enzyme. This gives the opportunity to the molecules to develop a vast number of interactions between the amino acids of the active site and their pharmacophore groups. Green arrows symbolize π-π stacking interactions, purple arrows symbolize hydrogen bonds, while light blue, green, deep blue and red amino acid residues symbolize polar, hydrophobic, positively and negatively charged residues, respectively.

**Figure 6:**
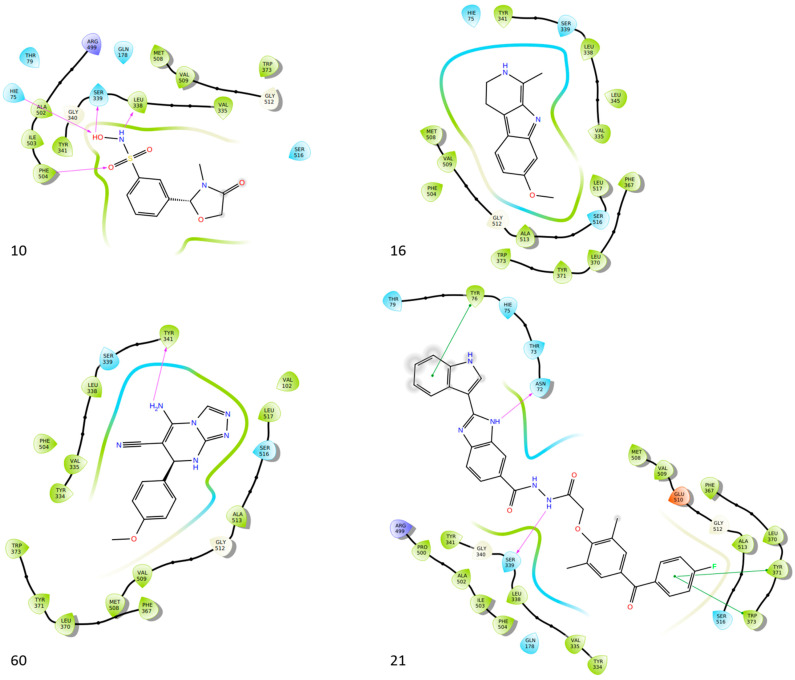
2D ligand interactions’ diagrams of studied compounds with the lowest affinity for the COX-2 enzyme (PDB ID: 3LN1). It can be observed that compounds **10**, **16** and **60** are too small, so they are not able to form the network of interactions that compounds **2**, **41**, **63** and **69** are able to achieve within the active pocket. An exception is compound **21** which develops a lot of interactions with the active site. Nonetheless, this must be the case of a false positive result of docking experiments, since the IC_50_ value of the compound is very high (39.43 ± 1.13 μM). Green arrows symbolize π-π stacking interactions, purple arrows symbolize hydrogen bonds, while light blue, green, deep blue and red amino acid residues symbolize polar, hydrophobic, positively and negatively charged residues, respectively.

The 2D ligand interactions’ diagrams can provide us with further information on the role of its pharmacophore group we are presenting in our rules. As it can be seen in Figure 5 and Figure 6, compounds **2**, **41**, **63** and **69** are able to indeed interact with more amino acid residues of the active site of COX-2 enzyme when compared to compounds **10**, **16** and **60** which showcase higher binding energies and IC_50_ values. This is achieved thanks to their extended carbon backbone. The only example of a compound showcasing a different in vitro profile than the one expected (showing a high IC_50_ value), is compound **21** and the reasoning behind the why this happens has been analyzed previously (overextension of the backbone results in poorer affinity for the target). The mobility of the bonds in the molecules also gives them an improved ability to interact with the cavity of the active site. The aromatic nature of the compounds, bearing more than one benzyl rings, gives them the ability to interact via π-π stacking with aromatic amino acids of the cavity, further stabilizing the complex, as can be seen with compound **41**. The existence of the heterocyclic five membered ring further stabilizes the complex through polar interactions with polar amino acid residues. Finally, the sulfonamide group due to its size capability to form hydrogen bonds with polar amino acids, further stabilizes the complex. For example, let’s compare the interaction profile between the sulfonamide group of compound **2** and the 1,4-Naphthoquinone group of compound **63**. In both cases hydrogen bonds are formed, but in the case of compound **2** two hydrogen bonds are formed, while in the case of **63** only one hydrogen bond is observed.

**Figure 7:**
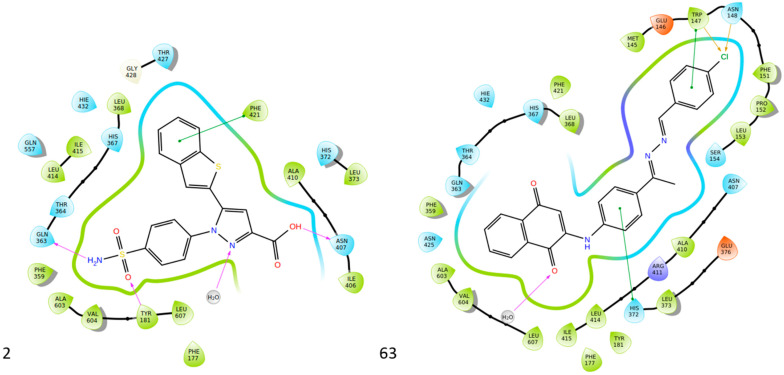
2D ligand interactions’ diagrams of studied compounds with the highest affinity for the 5-LOX enzyme (PDB ID: 3O8Y). It can be observed that these compounds (**2** and **63**) are able to penetrate deeply into the active site cavity of the 5-LOX enzyme. This gives the opportunity to the molecules to develop a vast number of interactions between the amino acids of the active site and their pharmacophore groups. Green arrows symbolize π-π stacking interactions, purple arrows symbolize hydrogen bonds, while light blue, green, deep blue and red amino acid residues symbolize polar, hydrophobic, positively and negatively charged residues, respectively.

**Figure 8:**
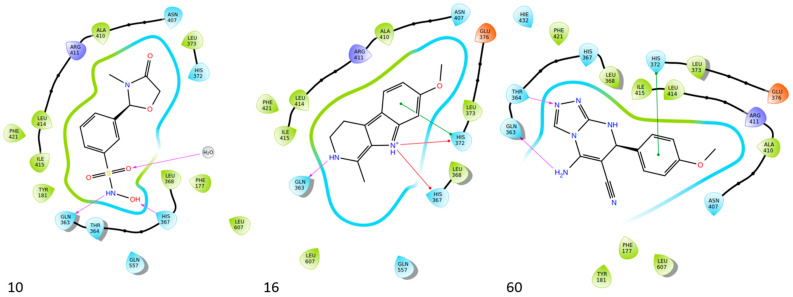
2D ligand interaction diagrams of studied compounds with the lowest affinity for the 5-LOX enzyme (PDB ID: 3O8Y). It can be observed that compounds **10**, **16** and **60** are too small in size, so they are not able to form the network of interactions that compounds **2** and **63** are able to achieve within the active pocket. Green arrows symbolize π-π stacking interactions, purple arrows symbolize hydrogen bonds, while light blue, green, deep blue and red amino acid residues symbolize polar, hydrophobic, positively and negatively charged residues, respectively.

For the 5-LOX enzyme, the trend remains the same as can be observed for the highest affinity compounds **2** and **63** and for the lowest affinity compounds **10**, **16** and **60**. Compounds **2** and **63** are able to interact with more amino acid residues of the active site cavity of 5-LOX enzyme (Figure 7) when compared to **10**, **16** and **60** (Figure 8). Unfortunately, due to computational restrictions, it seems that the Maestro software does not recognize compounds **41**, **69** and **21** as capable of entering the enzyme cavity of 5-LOX (N/A), thus not providing a binding energy for these molecular entities. This is probably due to their size. Nonetheless, the fact that they inhibit in vitro the enzymes does not detract from the conclusions we have already drawn.

Even when we compare the structure of celecoxib to the one of the newly synthesized and studied compounds **2**, **41**, **63** and **69** we can observe a lot of similarities. Celecoxib processes all four characteristics of “*The Rule of Four for Inflammation*”. Its IC_50_ values and binding energies are closely related to those of the studied compounds, with the studied compounds outperforming celecoxib. The same can be applied for zileuton. In this case zileuton complies with all proposed rules except rules 1 and 4. It is a relatively small molecule which has a five-membered heterocyclic ring not capable of rotating and misses its sulfonamide group (though it possesses an amide group). It shows high binding affinity for the target, but if optimized with our proposed model, it could result in molecules with better affinity for the 5-LOX enzyme. Of course, if both drugs were optimized, dual inhibition of both COX-2 and 5-LOX could be achieved.

**Figure 9:**
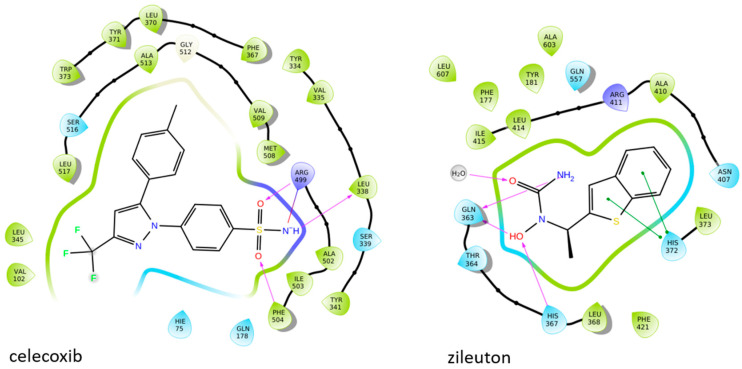
2D ligand interactions’ diagrams of studied drugs celecoxib and zileuton with COX-2 (PDB ID: 3LN1) and 5-LOX (PDB ID: 3O8Y) enzymes, respectively. It can be observed that celecoxib interacts with a multitude of amino acid residues. Zileuton, even though not interacting with the same amount of residues, it develops multiple π-π stacking interactions and hydrogen bonds, further adding to its inhibitory potency. Green arrows symbolize π-π stacking interactions, purple arrows symbolize hydrogen bonds, while light blue, green and deep blue and red amino acid residues symbolize polar, hydrophobic and positively charged residues, respectively.

As can be observed from the 2D-ligand interactions’ diagrams in Figure 9, celecoxib develops multiple interactions with the active site cavity of COX-2. On the other hand, zileuton, even though it does not have the multitude of interactions that celecoxib possesses, is capable of forming multiple π-π stacking and hydrogen bond interactions with the amino acid residues of the active site of 5-LOX. Based on our previous comments, lengthening the structure of zileuton, while adding more rotational degrees of freedom into the system and retaining its aromaticity, could greatly add to the compound’s potency.

### 3.3. Comparative Analysis of the Active Sites of COX-2/5-LOX

**Figure 10:**
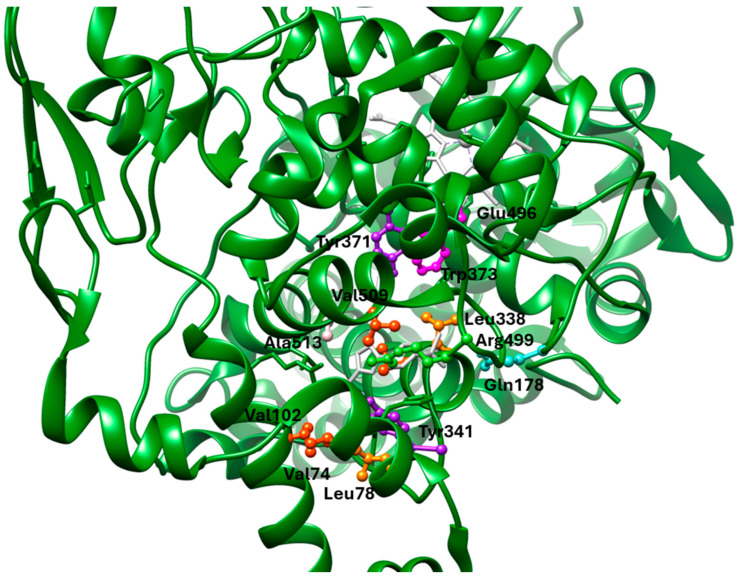
Active site of human cyclooxygenase-2 (COX-2, PDB ID: 3LN1). Key active-site residues are depicted in ball and stick representation, color-coded for clarity: Val74, Val102, Val509 (orange), Tyr341, Tyr371 (dark purple), Trp373 (magenta), Glu496 (purple), Gln178 (turquoise), Ala513 (white), and Leu78, Leu338 (light orange), Arg499 (light green). These residues are primarily involved in the coordination of the iron cofactor and substrate binding.

**Figure 11:**
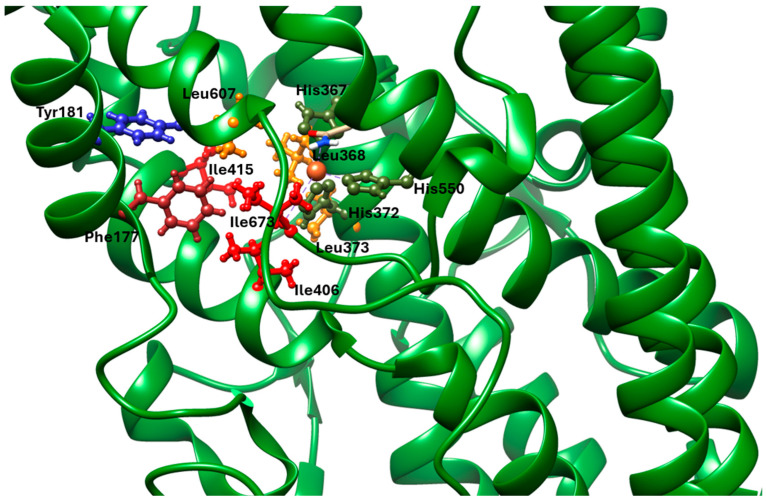
Active site of human 5-lipoxygenase (5-LOX, PDB ID: 3O8Y). The structure highlights key active-site residues involved in substrate and inhibitor binding. Residues are shown in ball and stick representation and colored by group: Tyr181 (blue), Phe177 (brown), Ile415, Ile406, Ile673 (red), Leu607, Leu368, Leu373 (light orange) and His367, His372, His550 (olive green). These residues form the catalytic core and the hydrophobic binding pocket of the enzyme.

A comparative analysis (Table 3) of the active sites of COX-2 and 5-LOX reveals limited overlap in the types of amino acids involved in catalysis and substrate binding (Figure 10 and Figure 11). Both enzymes share hydrophobic residues such as Leu, which likely contribute to forming nonpolar pockets accommodating fatty acid substrates. The presence of Tyr residues in both enzymes suggests a conserved role in stabilizing the substrate or intermediates via hydrogen bonding or π-π interactions.

Additionally, aromatic residues such as Trp and Phe appear uniquely in each enzyme, possibly reflecting differing strategies for substrate recognition and positioning.

These findings highlight both evolutionary divergence and functional specialization of these enzymes within the AA metabolism pathway, reinforcing the rationale for selective dual inhibitors of the two enzymes.

### 3.4. Machine Learning Classification

**Figure 12:**
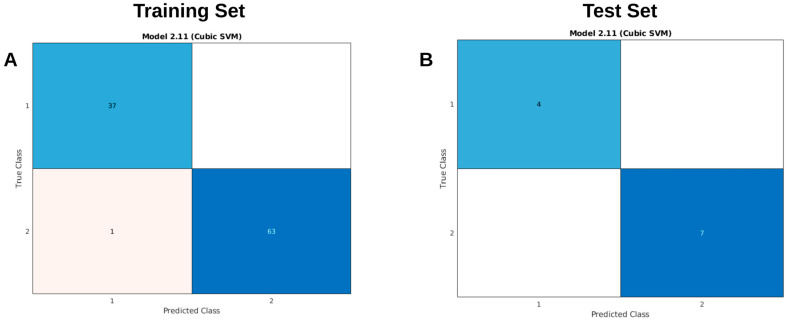
Machine learning classification based on 277 molecular descriptors, calculated from Molecular Descriptors workflow as implemented on Schrödinger Suite. The Cubic Support Vector Machine gave 99.1% prediction accuracy (**A**) for the training set while the algorithm achieved 100% accuracy on test set (**B**).

We selected 31 compounds determined as potent COX/LOX dual inhibitors (compounds from Table 1) and 19 compounds which are determined experimentally as weak dual inhibitors (based on the bibliography from Table 1). For all selected compounds, we run the ligprep workflow as implemented in Schrödinger Suite resulting 112 compounds (tautomers and different protonation states). Then we split the dataset to training set (101) compounds and test set (11 compounds). 277 molecular descriptors were calculated from Molecular Descriptors workflow as implemented on Schrödinger Suite. MATLAB v2018b Classification Learner was utilized to select the most accurate machine learning algorithm for discrimination based on the prediction accuracy resulted. All mathematical calculations were run using the software package MATLAB v2018b developed by MathWorks, Portola Valley, CA, USA (http://www.mathworks.com, accessed on 21 July 2021). The Cubic Support Vector Machine gave 99.1% prediction accuracy, (Figure 12A) for the training set while the algorithm achieved 100% accuracy on test set (Figure 12B).

### 3.5. Pharmacophore Model

**Figure 13:**
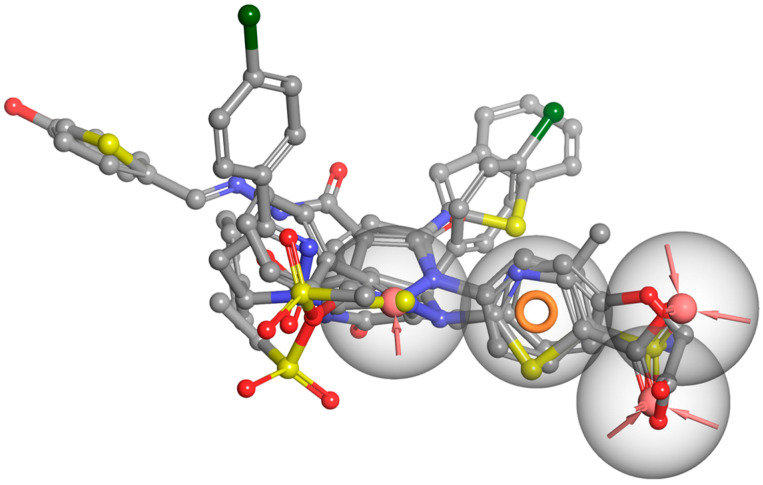
Pharmacophore model developed using Maestro software version 2021-02. The important pharmacophore- moieties were derived from the superimposition of the dual inhibitors from Table 1. Newly discovered compounds should possess these structural elements in order to exhibit potent inhibitory activity against COX-2/5-LOX. Specifically, they should contain 3 H-Bonds acceptors (pink spheres) and 1 aromatic ring (orange circle).

Furthermore, a preliminary pharmacophore model (Figure 13) was developed utilizing phase software as implemented on Maestro, Schrödinger. The dataset was divided to dual inhibitors (41 compounds) and non-dual inhibitors (71 compounds). The classification of dual and non-dual inhibitors was based on the references from Table 1. The phase algorithm resulted 6 hypothesis. The most dominant hypothesis is presented on Figure 13. The hypothesis is consisted by 3 H-Bonds acceptors (pink spheres) and 1 aromatic ring (orange circles).

### 3.6. Future Perspectives

**Figure 14:**
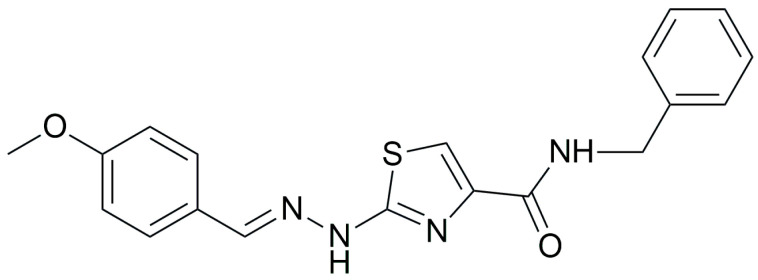
Structure of compound **75**. This molecule complies with all rules of “*The Rule of Four for Inflammation*”, with the difference that it does not contain the five-membered heterocyclic ring at the end of the structure and it possess an amide-moiety instead of a sulfonamide-one.

From the above analysis and structure–activity relationship (SAR) studies we have derived our proposed rule; “*The Rule of Four for Inflammation*”. During this study we wished to test some of our own compounds, which were designed before conducting this analysis, as COX-2 and 5-LOX inhibitors. Thus, we present below one molecule we studied in silico; compound **75** (Figure 14). Once more we utilized Maestro software of Schrödinger [127]. The ligand-protein complex showed a promising docking energy of −10.114 kcal/mol for COX-2 inhibition and a −11.210 kcal/mol for 5-LOX inhibition.

**Figure 15:**
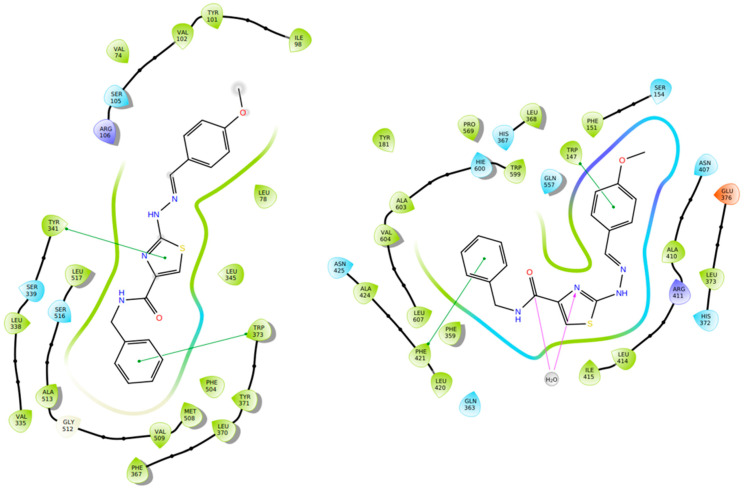
2D ligand interactions’ diagrams of compound **75** with COX-2 (PDB ID: 3LN1) and 5-LOX (PDB ID: 3O8Y) enzymes, respectively. Green arrows symbolize π-π stacking interactions, purple arrows symbolize hydrogen bonds, while light blue, green and deep blue and red amino acid residues symbolize polar, hydrophobic and positively charged residues, respectively.

As it can be observed from Figure 15, the structure of compound **75** complies with all necessary characteristics for a molecule to be a potent inhibitor of COX-2 and 5-LOX. It has a backbone of considerable length (around 18 carbon atoms), integrates multiple rotatable bonds, is an extended aromatic system and also has a five-membered heterocyclic ring in its structure. The only differences are that it does not contain the five-membered heterocyclic ring at the end of the structure and that it possesses an amide- moiety instead of a sulfonamide- one. Nonetheless, it shows good affinity with both enzymes and confirms our theory of “*The Rule of Four for Inflammation*”. In Figure 15 multiple interactions can be observed between the ligand and the protein-targets, further showcasing the ability of such molecules to strongly bind to these receptors.

## 4. Conclusions

The AA pathway is central to producing major inflammatory and signaling molecules, including PGs, LTs, and thromboxanes. When this pathway becomes deregulated, it can contribute to the development of various pathological conditions, including cardiovascular disorders, metabolic diseases, and cancer. Two major enzymes in this pathway—cyclooxygenases (COXs) and lipoxygenases (LOXs)—drive the formation of PGs and LTs, respectively. Because of their central roles in producing these inflammatory mediators, both enzyme families have long been viewed as important therapeutic targets for managing inflammatory disorders and other diseases linked to inflammation. In this review, we summarized the recent advances from the past four years concerning the AA pathway. We focused on the structure and biological functions of COX-1, COX-2, and 5-LOX, highlighting their involvement in inflammation and outlining the features of their active sites. We then discussed the most potent dual COX-2/5-LOX inhibitors reported between 2020 and 2024, drawing on findings from both in vitro and in vivo studies. From the structure–activity relationship (SAR) analysis of these molecules we concluded that there are 4 basic characteristics that must be met for a molecule to be a potent inhibitor of the cyclooxygenase-2 (COX-2) and 5-lipoxygenase (5-LOX) enzymes. We named these observations “*The Rule of Four for Inflammation*”. Firstly, these molecules need to have a relatively extended carbon backbone, spanning about 18 carbon atoms. Also, the existence of rotatable bonds, providing higher degrees of freedom for the molecules, is of the utmost importance. Moreover, a compound needs to be an extended aromatic system with multiple benzene rings. Finally, the existence of a heterocyclic five-membered ring (pyrazole-like) immediately connected via its 4-position with a benzenesulfonamide group, as well as being substituted with an amine- or methyl-group at its 5-position gives the molecule the chance to interact more potently with the active site of the aforementioned enzymes. As indicated by a preliminary pharmacophore model we have developed, the existence of the aromatic ring in the end of the chain, as well as 2 hydrogen bond acceptors near it and 1 hydrogen bond acceptor in the middle of the structure, further agree with our proposed theory. Computational studies further prove this model. Of course, more studies are needed to assess if this proposed rule can hold for more molecules of the bibliography or if further optimizations of the model are needed. Nevertheless, the foundations for the evaluation of this rule have been laid and we hope that it will be a powerful weapon in the quiver of pharmaceutical chemists to design and synthesize even more potent inhibitors of COX-2 and 5-LOX and consequently drugs.

## Figures and Tables

**Table 1:** In vitro inhibitory activity of selected synthetic and natural compounds with dual COX-2 and 5-LOX inhibition. The compound number in the text is shown in bold while the original compound number in the reference is shown in parentheses.

No. (Ref. No.)	Compound	COX-2 (IC_50_ μM)	5-LOX (IC_50_ μM)	Ref.
*2020*				
**1** (13e)	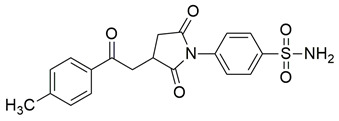	0.98 ± 0.01	0.86 ± 0.01	[88]
**2** (5b)	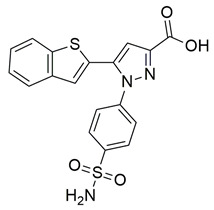	0.01 ± 0.001	1.78	[89]
**3** (6b)	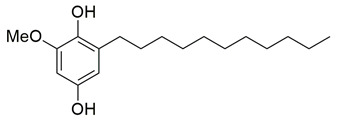	0.55 ± 0.19	0.28 ± 0.20	[90]
**4** (7h)	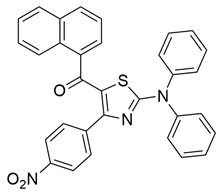	0.07 ± 0.02	0.29 ± 0.09	[91]
**5** (11)	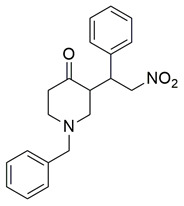	5.79 ± 0.23	1.06 ± 0.02	[92]
**6** (5b)	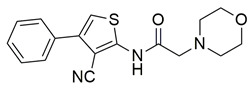	5.45 ± 0.13	4.33 ± 0.08	[93]
*2021*				
**7** (6l)	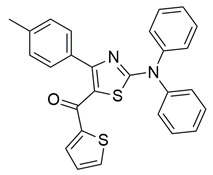	0.09 ± 0.002	0.38 ± 0.01	[94]
**8** (78)	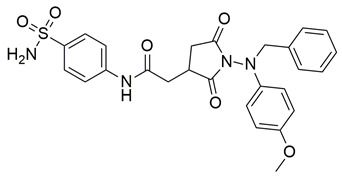	0.051	0.99 ± 0.10	[95]
**9** (5g)	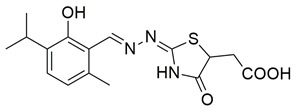	0.091 ± 0.0016	3.54 ± 0.075	[96]
**10** (1j)	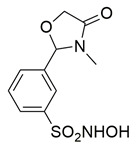	0.794	0.692	[97]
**11** (21)	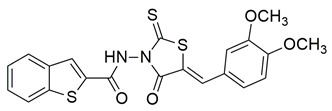	0.67	2.33	[98]
**12** (Erectascalarane A)	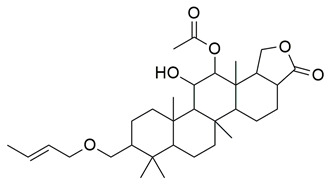	800 ± 20	1210 ± 20	[99]
**13** (3q)	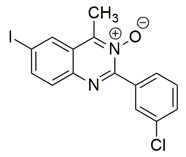	4.6 ± 1.45	15.0	[100]
**14** (38)	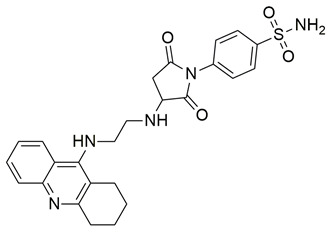	0.029 ± 0.003	0.54 ± 0.0001	[101]
*2022*				
**15** (6k)	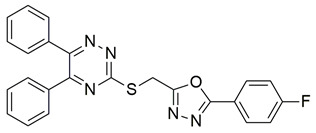	0.33 ± 0.02	4.90 ± 0.22	[102]
**16** (3)	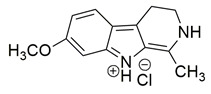	2.638 ± 0.07	1.63 ± 0.07	[103]
**17** (Compound 1)	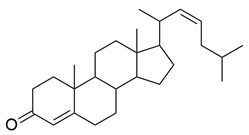	53	19	[104]
**18** (39)	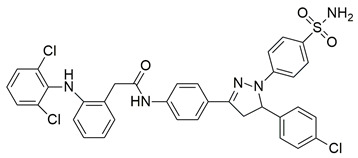	0.60 ± 0.03	0.98 ± 0.01	[105]
**19** (d)	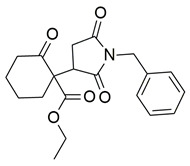	120	336	[106]
**20** (FM12)	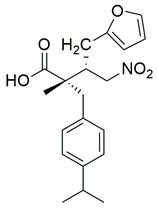	0.18 ± 0.01	0.43 ± 0.02	[107]
**21** (10c)	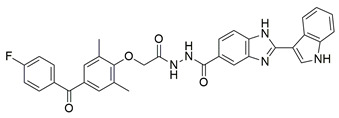	39.43 ± 1.13	1.78 ± 0.09	[108]
*2023*				
**22** (1)	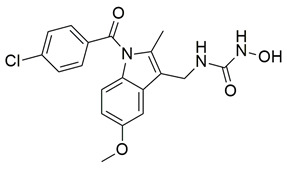	18.28 ± 2.17	5.71 ± 0.15	[109]
**23** (2)	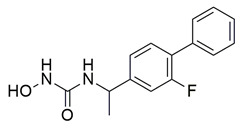	6.72 ± 0.79	1.62 ± 0.67	[109]
**24** (3)	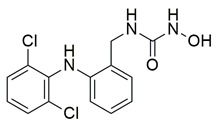	5.26 ± 0.34	1.73 ± 0.64	[109]
**25** (5)	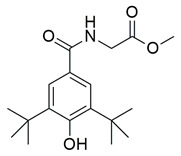	10.64 ± 0.80	9.30 ± 1.87	[109]
**26** (6)	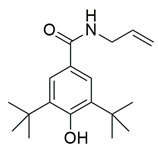	6.89 ± 0.83	53.84 ± 11.87	[109]
**27** (11)	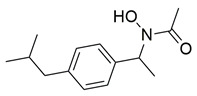	36.18 ± 3.08	1.04 ± 0.22	[109]
**28** (12)	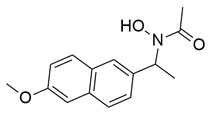	83.42 ± 4.37	1.29 ± 0.10	[109]
**29** (7f)	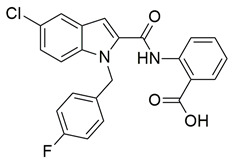	0.54 ± 0.033	0.077 ± 0.0015	[110]
**30** (7n)	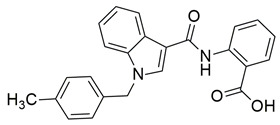	0.32 ± 0.039	0.22 ± 0.021	[110]
**31** (4a)	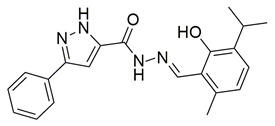	0.068 ± 0.01	3.05 ± 0.067	[111]
**32** (8b)	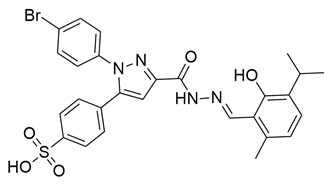	0.043 ± 0.001	1.58 ± 0.026	[111]
**33** (8c)	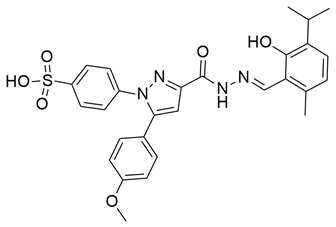	0.063 ± 0.001	1.91 ± 0.053	[111]
**34** (8g)	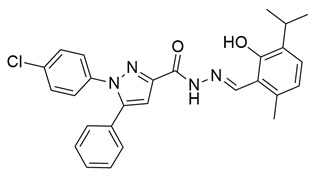	0.045 ± 0.01	1.60 ± 0.042	[111]
**35** (7a)	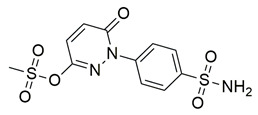	0.05	3	[112]
**36** (7b)	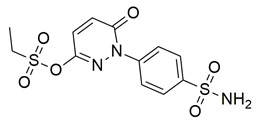	0.06	2.5	[112]
**37** (7a)	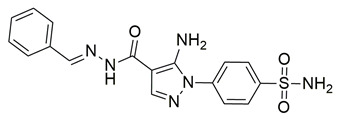	0.049 ± 0.001	2.4 ± 0.1	[113]
**38** (7b)	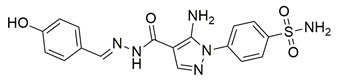	0.060 ± 0.002	1.9 ± 0.1	[113]
**39** (7j)	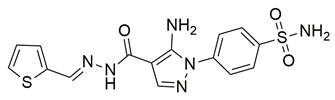	0.060 ± 0.001	2.5 ± 0.1	[113]
**40** (9a)	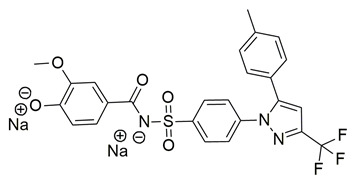	0.011	0.46	[114]
**41** (9b)	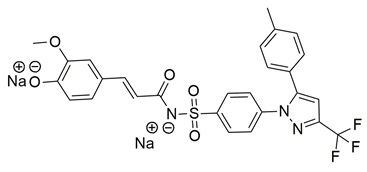	0.023	0.31	[114]
**42** (9c)	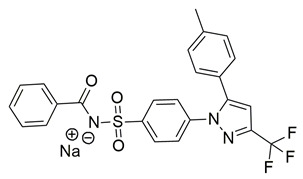	0.076	0.12	[114]
**43** (9e)	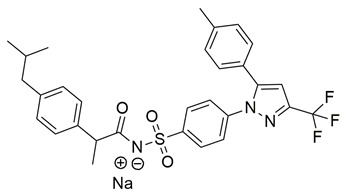	0.025	0.52	[114]
**44** (4e)	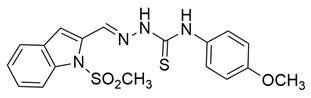	0.819 ± 0.04	4.08 ± 0.22	[115]
**45** (5d)	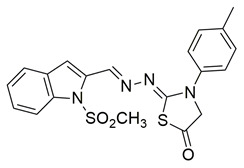	0.67 ± 0.04	1.10 ± 0.06	[115]
**46** (7c)	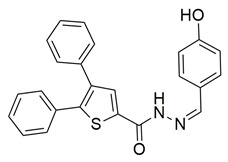	10.13 ± 0.25	2.60 ± 0.11	[116]
**47** (7e)	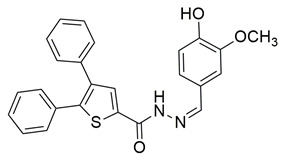	13.86 ± 0.76	3.30 ± 0.07	[116]
**48** (2)	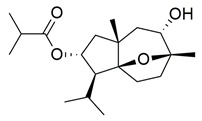	9.03 ± 1.196	0.56 ± 1.074	[117]
**49** (5a)	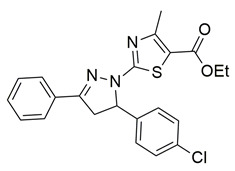	0.06 ± 0.004	4.36 ± 0.37	[118]
**50** (5b)	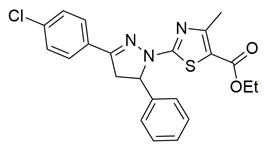	0.44 ± 0.015	2.40 ± 0.23	[118]
**51** (6a)	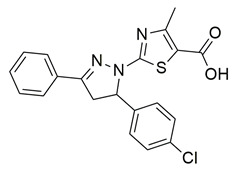	0.04 ± 0.004	4.86 ± 0.32	[118]
**52** (6b)	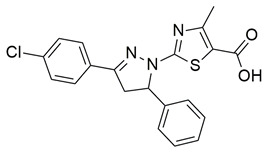	0.03 ± 0.003	4.86 ± 0.25	[118]
**53** (10a)	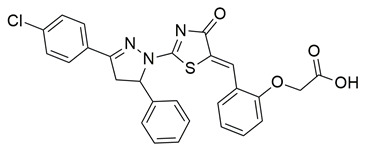	0.41 ± 0.04	1.58 ± 0.10	[118]
**54** (10c)	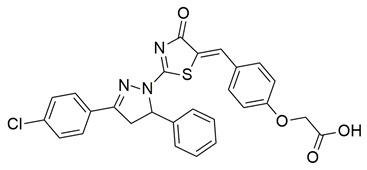	0.06 ± 0.003	3.54 ± 0.35	[118]
**55** (19)	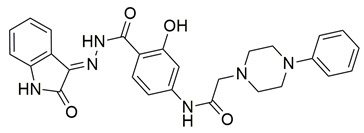	1.16 ± 0.05	4.38 ± 0.2	[119]
**56** (20)	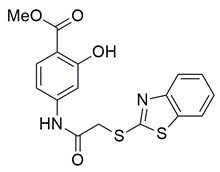	4.54 ± 0.21	9.64 ± 0.45	[119]
**57** (CW-D)	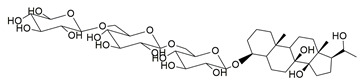	1.07 ± 0.01	26.30 ± 1.79	[120]
*2024*				
**58** (8b)	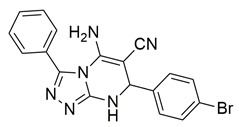	15.20	3.50 ± 0.04	[121]
**59** (8m)	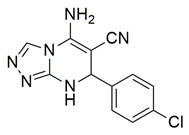	11.60	3.05 ± 0.03	[121]
**60** (8o)	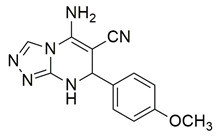	10.50	2.90 ± 0.03	[121]
**61** (5b)	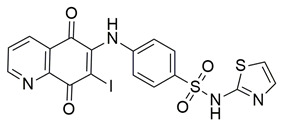	0.041	1.96	[122]
**62** (5d)	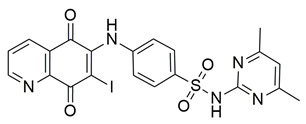	0.042	1.59	[122]
**63** (9a)	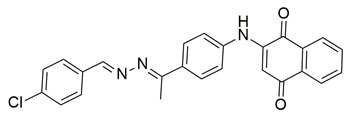	0.041	1.74	[122]
**64** (9b)	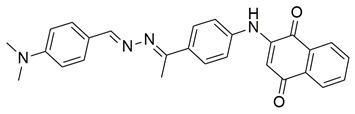	0.044	2.66	[122]
**65** (11b)	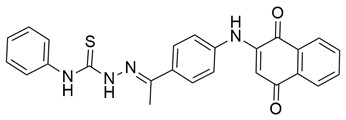	0.078	3.11	[122]
**66** (6b)	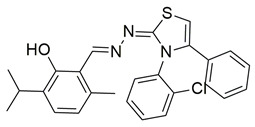	0.037 ± 0.007	2.51 ± 0.015	[123]
**67** (6d)	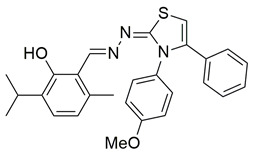	0.042 ± 0.001	2.47 ± 0.06	[123]
**68** (6e)	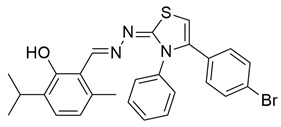	0.046 ± 0.0016	1.75 ± 0.06	[123]
**69** (6f)	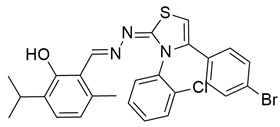	0.039 ± 0.001	1.53 ± 0.02	[123]
**70** (7f)	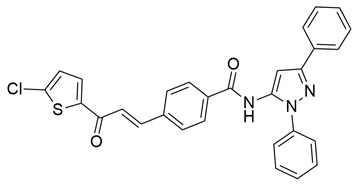	1.76 ± 0.03	0.27 ± 0.06	[124]
**71** (7g)	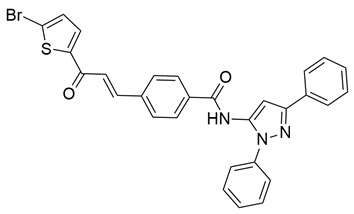	1.01 ± 0.02	0.29 ± 0.06	[124]
**72** (5d)	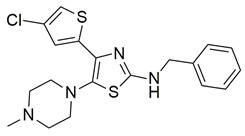	0.83 ± 0.03	23.08 ± 0.18	[125]
**73** (5e)	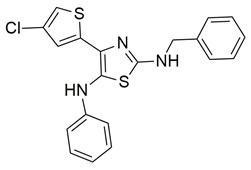	0.76 ± 0.17	38.45 ± 0.20	[125]
**74** (HSA-8)	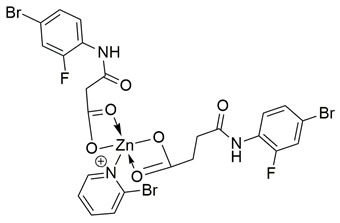	0.288	0.83	[126]

**Table 2:** In vitro and in silico inhibitory activity of selected synthetic and natural compounds with dual COX-2 and 5-LOX inhibition. These compounds were selected as they possessed the most balanced and highest affinity pharmacological profile towards the studied drug targets. They are categorized according to the affinities for COX-2/5-LOX and their agreement with “*The Rule of Four for Inflammation*”.

No.	Compound Structure	COX-2 (IC_50_ μM)	COX-2 Binding Energy (kcal/mol)	5-LOX (IC_50_ μM)	5-LOX Binding Energy (kcal/mol)
**Highest affinity for COX-2/5-LOX**					
*Group A; Full agreement with* “*The Rule of Four for Inflammation*”					
With the sulfonamide group at the end of the structure					
**2** (5b)	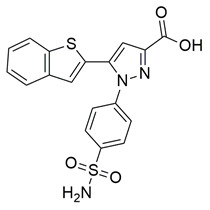	0.01 ± 0.001	−11.863	1.78	−10.632
With the sulfonamide group at the middle of the structure					
**41** (9b)	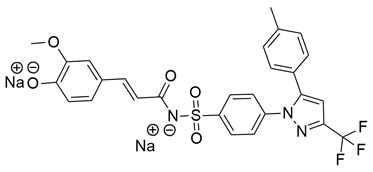	0.023	−12.569	0.31	N/A
Absence of the sulfonamide group					
**69** (6f)	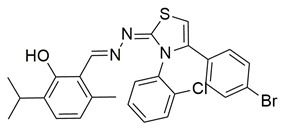	0.039 ± 0.001	−12.674	1.53 ± 0.02	N/A
*Group B; 3/4 agreement with* “*The Rule of Four for Inflammation*” *(absence of the fourth rule)*					
**63** (9a)	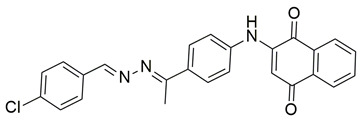	0.041	−9.067	1.74	−11.861
**Lowest affinity for COX-2/5-LOX**					
*Group A’; 1/4 agreement with* “*The Rule of Four for Inflammation*” *(absence of the first, second and third rule)*					
**10** (1j)	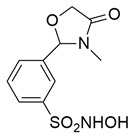	0.794	−8.074	0.692	−7.919
*Group Β’; 1/4 agreement with* “*The Rule of Four for Inflammation*” *(absence of the first, second and fourth rule)*					
**16** (3)	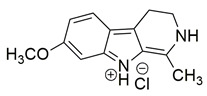	2.638 ± 0.07	−7.609	1.63 ± 0.07	−8.803
*Group C’; 2/4 agreement with* “*The Rule of Four for Inflammation*” *(absence of the first and fourth rule)*					
**60** (8o)	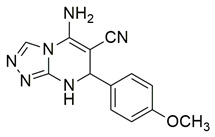	10.50	−8.789	2.90 ± 0.03	−7.629
*Group D’; 3/4 agreement with* “*The Rule of Four for Inflammation*” *(absence of the first rule)*					
**21** (10c)	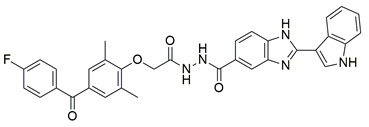	39.43 ± 1.13	−12.526	1.78 ± 0.09	N/A
**The example of the well-established drugs celexoxib (COX-2 inhibitor) and zileuton (5-LOX inhibitor)**					
**Celecoxib**	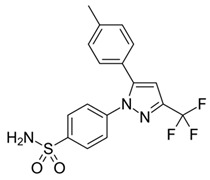	0.05 ± 0.01	−12.046	–	–
**Zileuton**	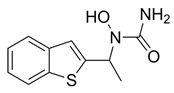	–	–	0.64 ± 0.06	−10.018

**Table 3:** Comparative analysis of amino acid types found in the active sites of cyclooxygenase-2 (COX-2, PDB ID: 3LN1) and 5-lipoxygenase (5-LOX, PDB ID: 3O8Y). The table lists the amino acid types that are present in the active-site regions visualized in the structural representations of each enzyme. Shared residue types between the two active sites are indicated, as well as those unique to each enzyme. Residue numbering is omitted to focus on chemical identity relevant to catalytic or substrate-binding roles.

Amino Acid Type	Present in 5-LOX (3O8Y)	Present in COX-2 (3LN1)	Shared/Unique
**Tyr (Tyrosine)**	Tyr181	Tyr341, Tyr371	Shared
**Phe (Phenylalanine)**	Phe177	—	Unique to 5-LOX
**His (Histidine)**	His367, His372, His550	—	Unique to 5-LOX
**Leu (Leucine)**	Leu368, Leu373, Leu607	Leu78, Leu338	Shared
**Ile (Isoleucine)**	Ile406, Ile415, Ile673	—	Unique to 5-LOX
**Val (Valine)**	—	Val74, Val102, Val509	Unique to COX-2
**Trp (Tryptophan)**	—	Trp373	Unique to COX-2
**Glu (Glutamic acid)**	—	Glu496	Unique to COX-2
**Arg (Arginine)**	—	Arg499	Unique to COX-2
**Gln (Glutamine)**	—	Gln178	Unique to COX-2
**Ala (Alanine)**	—	Ala513	Unique to COX-2

## Data Availability

No new data were created or analyzed in this study. Data sharing is not applicable to this article.

## Abbreviations

The following abbreviations are used in this manuscript:

AA	Arachidonic acid
COXs	Cyclooxygenases
PGs	Prostaglandins
TXA_2_	Thromboxane A_2_
LOXs	Lipoxygenases
LTs	Leukotrienes
AD	Alzheimer’s disease
AT1	Angiotensin II type 1 receptor
Aβ	amyloid beta
MTDLs	Multi-directed ligands
SAR	Structure–activity relationship
COX-2	Cyclooxygenase-2
5-LOX	5-lipoxygenase
PUFA	Polyunsaturated fatty acid
PLA2	Phospholipase A2
PLC	Phospholipase C
PLD	Phospholipase D
PGG_2_	Prostaglandin G_2_
PGH_2_	Prostaglandin H_2_
PGS	Prostaglandin synthases
ALOX5	5-lipoxygenase
CYP	Cytochrome P450
HETEs	Hydroxyeicosatetraenoic acids
EETs	epoxyeicosatrienoic acids
sEH	Soluble epoxide hydrolase
DHETEs	Dihydroxyeicosatrienoic acids
CVDs	Cardiovascular diseases
cPGES	cytosolic PGE synthase
PGIS	Prostacyclin synthase
mPGES	microsomal PGE synthase
5-HPETE	5-hydroperoxyeicosatetraenoate
LXs	Lipoxins
PGHS	Prostaglandin G/H synthases
LTS	Leukotriene synthase
PMNs	Polymorphonuclear leukocytes
PPAR_alpha_	Peroxisome proliferator-activated receptor alpha
PPAR_Gamma_	Peroxisome proliferator-activated receptor gamma
EGF	Epidermal growth factor
FGF	Fibroblast growth factor
VEGF	Vascular endothelial growth factor
TNF	Tumor necrosis factor
NSAIDs	Nonsteroidal anti-inflammatory drugs
GPX	Glutathione peroxidase
5-LO	5-lipoxygenase
LTA_4_	Leukotriene A_4_
LXA4	Lipoxin A4
LXB4	Lipoxin B4
IC_50_	Half-maximal inhibitory concentration
DPPH	2,2-diphenyl-1-picrylhydrazyl
RMSD	Root mean square deviation
RMSF	Root mean square fluctuation
ELISA	Enzyme-linked immunosorbent assay
ADME	Absorption, distribution, metabolism, and excretion
TNF-α	Tumor necrosis factor alpha
IL-1β	Interleukin-1β
IL-6	Interleukin-6
LPS	Lipopolysaccharide
BV-2	Immortalized microglial cell line
MM-GBSA	Molecular Mechanics/Generalized Born Surface Area

## References

[1] Alharbi K.S., Alenezi S.K., Gupta G. (2023). Pathophysiology and Pathogenesis of Inflammation. Recent Developments in Anti-Inflammatory Therapy.

[2] Wang B., Wu L., Chen J., Dong L., Chen C., Wen Z., Hu J., Fleming I., Wang D.W. (2021). Metabolism Pathways of Arachidonic Acids: Mechanisms and Potential Therapeutic Targets. Signal Transduct. Target. Ther..

[3] Ricciotti E., FitzGerald G.A. (2011). Prostaglandins and Inflammation. Arterioscler. Thromb. Vasc. Biol..

[4] Eckenstaler R., Benndorf R.A. (2025). Insights into the Expression, Structure, and Function of the Thromboxane A_2_ Receptor in Vascular Biology. ACS Pharmacol. Transl. Sci..

[5] Henderson W.R. (1994). The Role of Leukotrienes in Inflammation. Ann. Intern. Med..

[6] Zhou Y., Khan H., Xiao J., Cheang W.S. (2021). Effects of Arachidonic Acid Metabolites on Cardiovascular Health and Disease. Int. J. Mol. Sci..

[7] Korbecki J., Rębacz-Maron E., Kupnicka P., Chlubek D., Baranowska-Bosiacka I. (2023). Synthesis and Significance of Arachidonic Acid, a Substrate for Cyclooxygenases, Lipoxygenases, and Cytochrome P450 Pathways in the Tumorigenesis of Glioblastoma Multiforme, Including a Pan-Cancer Comparative Analysis. Cancers.

[8] Donowitz M. (1985). Arachidonic Acid Metabolites and Their Role in Inflammatory Bowel Disease. Gastroenterology.

[9] Luo Y., Jin M., Lou L., Yang S., Li C., Li X., Zhou M., Cai C. (2022). Role of Arachidonic Acid Lipoxygenase Pathway in Asthma. Prostaglandins Other Lipid Mediat..

[10] Zhu H., Shen F., Liao T., Qian H., Liu Y. (2024). Sporidiobolus Pararoseus Polysaccharides Relieve Rheumatoid Arthritis by Regulating Arachidonic Acid Metabolism and Bone Remodeling Signaling Pathway. Int. J. Biol. Macromol..

[11] Zhang Y., Liu Y., Sun J., Zhang W., Guo Z., Ma Q. (2023). Arachidonic Acid Metabolism in Health and Disease. MedComm.

[12] Lyhne M.D., Kline J.A., Nielsen-Kudsk J.E., Andersen A. (2020). Pulmonary Vasodilation in Acute Pulmonary Embolism—A Systematic Review. Pulm. Circ..

[13] Erichev V.P. (2022). Prostaglandins in Ophthalmology. Vestn. Oftalmol..

[14] Nakamura M., Yokomizo T., Offermanns S., Rosenthal W. (2020). Leukotrienes. Encyclopedia of Molecular Pharmacology.

[15] Van Beusecum J.P., Barbaro N.R., McDowell Z., Aden L.A., Xiao L., Pandey A.K., Itani H.A., Himmel L.E., Harrison D.G., Kirabo A. (2019). High Salt Activates CD11c^+^ Antigen-Presenting Cells via SGK (Serum Glucocorticoid Kinase) 1 to Promote Renal Inflammation and Salt-Sensitive Hypertension. Hypertension.

[16] Wu J., Saleh M.A., Kirabo A., Itani H.A., Montaniel K.R.C., Xiao L., Chen W., Mernaugh R.L., Cai H., Bernstein K.E. (2015). Immune Activation Caused by Vascular Oxidation Promotes Fibrosis and Hypertension. J. Clin. Investig..

[17] Guzik T.J., Hoch N.E., Brown K.A., McCann L.A., Rahman A., Dikalov S., Goronzy J., Weyand C., Harrison D.G. (2007). Role of the T Cell in the Genesis of Angiotensin II–Induced Hypertension and Vascular Dysfunction. J. Exp. Med..

[18] Norlander A.E., Saleh M.A., Pandey A.K., Itani H.A., Wu J., Xiao L., Kang J., Dale B.L., Goleva S.B., Laroumanie F. (2017). A Salt-Sensing Kinase in T Lymphocytes, SGK1, Drives Hypertension and Hypertensive End-Organ Damage. JCI Insight.

[19] Patrick D.M., Van Beusecum J.P., Kirabo A. (2021). The Role of Inflammation in Hypertension: Novel Concepts. Curr. Opin. Physiol..

[20] Chatzipieris F.P., Kokkalis A., Georgiou N., Petsas E., Apostolou E.V., Vougioukalakis G.C., Mavromoustakos T. (2025). New Prospects in the Inhibition of Monoamine Oxidase-B (MAO-B) Utilizing Propargylamine Derivatives for the Treatment of Alzheimer’s Disease: A Review. ACS Omega.

[21] Chatzipieris F.P., Mavromoustakou K., Matsoukas J.M., Mavromoustakos T. (2025). Unlocking Novel Therapeutic Potential of Angiotensin II Receptor Blockers. Int. J. Mol. Sci..

[22] Chatzipieris F.P., Petsas E., Lambrinidis G., Matsoukas J.M., Mavromoustakos T. (2025). Structural and Computational Insights into the Angiotensin II Type 1 Receptor: Advances in Antagonist Design and Implications for Hypertension Therapy (2020–2024). Biomolecules.

[23] Süleyman H., Demircan B., Karagöz Y. (2007). Anti-Inflammatory and Side Effects of Cyclooxygenase Inhibitors. Pharmacol. Rep..

[24] Dahlke P., Peltner L.K., Jordan P.M., Werz O. (2023). Differential Impact of 5-Lipoxygenase-Activating Protein Antagonists on the Biosynthesis of Leukotrienes and of Specialized pro-Resolving Mediators. Front. Pharmacol..

[25] Seccia T.M., Caroccia B., Maiolino G., Cesari M., Rossi G.P. (2019). Arterial Hypertension, Aldosterone, and Atrial Fibrillation. Curr. Hypertens. Rep..

[26] De Mello W.C. (2017). Local Renin Angiotensin Aldosterone Systems and Cardiovascular Diseases. Med. Clin. N. Am..

[27] McKinney C.A., Fattah C., Loughrey C.M., Milligan G., Nicklin S.A. (2014). Angiotensin-(1–7) and Angiotensin-(1–9): Function in Cardiac and Vascular Remodelling. Clin. Sci..

[28] Senbonmatsu T. (2022). Prorenin: What Are Its Functions?. Hypertens. Res..

[29] Panigrahy D., Kaipainen A., Greene E.R., Huang S. (2010). Cytochrome P450-Derived Eicosanoids: The Neglected Pathway in Cancer. Cancer Metastasis Rev..

[30] Ni K.-D., Liu J.-Y. (2021). The Functions of Cytochrome P450 ω-Hydroxylases and the Associated Eicosanoids in Inflammation-Related Diseases. Front. Pharmacol..

[31] Fleming I. (2001). Cytochrome P450 and Vascular Homeostasis. Circ. Res..

[32] De Gaetano G., Donati M.B., Cerletti C. (2003). Prevention of Thrombosis and Vascular Inflammation: Benefits and Limitations of Selective or Combined COX-1, COX-2 and 5-LOX Inhibitors. Trends Pharmacol. Sci..

[33] Adili R., Tourdot B.E., Mast K., Yeung J., Freedman J.C., Green A., Luci D.K., Jadhav A., Simeonov A., Maloney D.J. (2017). First Selective 12-LOX Inhibitor, ML355, Impairs Thrombus Formation and Vessel Occlusion In Vivo With Minimal Effects on Hemostasis. Arterioscler. Thromb. Vasc. Biol..

[34] Rudrapal M., Eltayeb W.A., Rakshit G., El-Arabey A.A., Khan J., Aldosari S.M., Alshehri B., Abdalla M. (2023). Dual Synergistic Inhibition of COX and LOX by Potential Chemicals from Indian Daily Spices Investigated through Detailed Computational Studies. Sci. Rep..

[35] Alvaro-Gracia J.M. (2004). Licofelone—Clinical Update on a Novel LOX/COX Inhibitor for the Treatment of Osteoarthritis. Rheumatology.

[36] Wang T., Fu X., Chen Q., Patra J.K., Wang D., Wang Z., Gai Z. (2019). Arachidonic Acid Metabolism and Kidney Inflammation. Int. J. Mol. Sci..

[37] Hanna V.S., Hafez E.A.A. (2018). Synopsis of Arachidonic Acid Metabolism: A Review. J. Adv. Res..

[38] Jang Y., Kim M., Hwang S.W. (2020). Molecular Mechanisms Underlying the Actions of Arachidonic Acid-Derived Prostaglandins on Peripheral Nociception. J. Neuroinflamm..

[39] Romanelli M.N. (2024). Cyclooxygenase. Metalloenzymes.

[40] Faki Y., Er A. (2021). Different Chemical Structures and Physiological/Pathological Roles of Cyclooxygenases. Rambam Maimonides Med. J..

[41] Vishnupriya P., Aparna A., Viswanadha V.P. (2021). Lipoxygenase (LOX) Pathway: A Promising Target to Combat Cancer. Curr. Pharm. Des..

[42] Yokomizo T., Nakamura M., Shimizu T. (2018). Leukotriene Receptors as Potential Therapeutic Targets. J. Clin. Investig..

[43] Diamant Z., Mantzouranis E., Bjermer L. (2009). Montelukast in the Treatment of Asthma and Beyond. Expert Rev. Clin. Immunol..

[44] Kumar A., Behl T., Jamwal S., Kaur I., Sood A., Kumar P. (2020). Exploring the Molecular Approach of COX and LOX in Alzheimer’s and Parkinson’s Disorder. Mol. Biol. Rep..

[45] Luo B., Yan D., Yan H., Yuan J. (2021). Cytochrome P450: Implications for Human Breast Cancer (Review). Oncol. Lett..

[46] Majewski M., Juśkiewicz J., Krajewska-Włodarczyk M., Gromadziński L., Socha K., Cholewińska E., Ognik K. (2021). The Role of 20-HETE, COX, Thromboxane Receptors, and Blood Plasma Antioxidant Status in Vascular Relaxation of Copper-Nanoparticle-Fed WKY Rats. Nutrients.

[47] Stipp M.C., Acco A. (2021). Involvement of Cytochrome P450 Enzymes in Inflammation and Cancer: A Review. Cancer Chemother. Pharmacol..

[48] Gui L., Xu Q., Huang J., Wu G., Tang H., Hui L., Hua P., Zhang L., Zhu Y. (2020). CYP2J2 Promotes the Development of Hepatocellular Carcinoma by Increasing the EETs Production to Improve HIF-1α Stability. Am. J. Transl. Res..

[49] Xu X., Zhang X.A., Wang D.W. (2011). The Roles of CYP450 Epoxygenases and Metabolites, Epoxyeicosatrienoic Acids, in Cardiovascular and Malignant Diseases. Adv. Drug Deliv. Rev..

[50] Imig J.D. (2018). Prospective for Cytochrome P450 Epoxygenase Cardiovascular and Renal Therapeutics. Pharmacol. Ther..

[51] Biringer R.G. (2020). The Enzymology of Human Eicosanoid Pathways: The Lipoxygenase Branches. Mol. Biol. Rep..

[52] Patrono C. (2024). Low-Dose Aspirin for the Prevention of Atherosclerotic Cardiovascular Disease. Eur. Heart J..

[53] Liu J., Seibold S.A., Rieke C.J., Song I., Cukier R.I., Smith W.L. (2007). Prostaglandin Endoperoxide H Synthases. J. Biol. Chem..

[54] Marnett L.J., Rowlinson S.W., Goodwin D.C., Kalgutkar A.S., Lanzo C.A. (1999). Arachidonic Acid Oxygenation by COX-1 and COX-2. J. Biol. Chem..

[55] Byrne M.F., Murphy J.F., Corcoran P.A., Atherton J.C., Sheehan K.M., Cox D., Murray F.E., Fitzgerald D.J. (2003). *Helicobacter pylori* Induces Cyclooxygenase-1 and Cyclooxygenase-2 Expression in Vascular Endothelial Cells. Scand. J. Gastroenterol..

[56] Cullen L., Kelly L., Connor S.O., Fitzgerald D.J. (1998). Selective Cyclooxygenase-2 Inhibition by Nimesulide in Man. J. Pharmacol. Exp. Ther..

[57] Smith W.L., Malkowski M.G. (2019). Interactions of Fatty Acids, Nonsteroidal Anti-Inflammatory Drugs, and Coxibs with the Catalytic and Allosteric Subunits of Cyclooxygenases-1 and -2. J. Biol. Chem..

[58] Kam P.C.A., So A. (2009). COX-3: Uncertainties and Controversies. Curr. Anaesth. Crit. Care.

[59] Gilbert N.C., Bartlett S.G., Waight M.T., Neau D.B., Boeglin W.E., Brash A.R., Newcomer M.E. (2011). The Structure of Human 5-Lipoxygenase. Science.

[60] Giménez-Bastida J.A., González-Sarrías A., Laparra-Llopis J.M., Schneider C., Espín J.C. (2021). Targeting Mammalian 5-Lipoxygenase by Dietary Phenolics as an Anti-Inflammatory Mechanism: A Systematic Review. Int. J. Mol. Sci..

[61] Trostchansky A., Wood I., Rubbo H. (2021). Regulation of Arachidonic Acid Oxidation and Metabolism by Lipid Electrophiles. Prostaglandins Other Lipid Mediat..

[62] Tomé-Rodríguez S., Ledesma-Escobar C.A., Penco-Valenzuela J.M., Priego-Capote F. (2021). Cultivar Influence on the Volatile Components of Olive Oil Formed in the Lipoxygenase Pathway. LWT.

[63] Thalanayar Muthukrishnan P., Nouraie M., Parikh A., Holguin F. (2020). Zileuton Use and Phenotypic Features in Asthma. Pulm. Pharmacol. Ther..

[64] Gong L., Thorn C.F., Bertagnolli M.M., Grosser T., Altman R.B., Klein T.E. (2012). Celecoxib Pathways: Pharmacokinetics and Pharmacodynamics. Pharmacogenet. Genom..

[65] Mukherjee D., Nissen S.E., Topol E.J. (2001). Risk of Cardiovascular Events Associated with Selective COX-2 Inhibitors. JAMA.

[66] McGill K.A., Busse W.W. (1996). Zileuton. Lancet.

[67] Bièche I., Narjoz C., Asselah T., Vacher S., Marcellin P., Lidereau R., Beaune P., De Waziers I. (2007). Reverse Transcriptase-PCR Quantification of mRNA Levels from Cytochrome (CYP)1, CYP2 and CYP3 Families in 22 Different Human Tissues. Pharmacogenet. Genom..

[68] Kuban W., Daniel W.A. (2021). Cytochrome P450 Expression and Regulation in the Brain. Drug Metab. Rev..

[69] Stanley L.A. (2024). Drug Metabolism. Pharmacognosy.

[70] Morgan E.T., Li-Masters T., Cheng P.-Y. (2002). Mechanisms of Cytochrome P450 Regulation by Inflammatory Mediators. Toxicology.

[71] Zhao M., Ma J., Li M., Zhang Y., Jiang B., Zhao X., Huai C., Shen L., Zhang N., He L. (2021). Cytochrome P450 Enzymes and Drug Metabolism in Humans. Int. J. Mol. Sci..

[72] Nayeem M.A., Geldenhuys W.J., Hanif A. (2023). Role of Cytochrome P450-Epoxygenase and Soluble Epoxide Hydrolase in the Regulation of Vascular Response. Advances in Pharmacology.

[73] Johnson A.L., Edson K.Z., Totah R.A., Rettie A.E. (2015). Cytochrome P450 ω-Hydroxylases in Inflammation and Cancer. Advances in Pharmacology.

[74] Drenjančević I., Jukić I., Mihaljević Z., Ćosić A., Kibel A., Lenasi H. (2016). The Metabolites of Arachidonic Acid in Microvascular Function. Microcirculation Revisited-From Molecules to Clinical Practice.

[75] Dennis E.A., Norris P.C. (2015). Eicosanoid Storm in Infection and Inflammation. Nat. Rev. Immunol..

[76] Luo P., Wang M.-H. (2011). Eicosanoids, β-Cell Function, and Diabetes. Prostaglandins Other Lipid Mediat..

[77] Neckář J., Kopkan L., Husková Z., Kolář F., Papoušek F., Kramer H.J., Hwang S.H., Hammock B.D., Imig J.D., Malý J. (2012). Inhibition of Soluble Epoxide Hydrolase by *Cis*-4-[4-(3-Adamantan-1-Ylureido)Cyclohexyl-Oxy]Benzoic Acid Exhibits Antihypertensive and Cardioprotective Actions in Transgenic Rats with Angiotensin II-Dependent Hypertension. Clin. Sci..

[78] Fan F., Muroya Y., Roman R.J. (2015). Cytochrome P450 Eicosanoids in Hypertension and Renal Disease. Curr. Opin. Nephrol. Hypertens..

[79] Rouzer C.A., Marnett L.J. (2020). Structural and Chemical Biology of the Interaction of Cyclooxygenase with Substrates and Non-Steroidal Anti-Inflammatory Drugs. Chem. Rev..

[80] Yadav M., Abdalla M., Madhavi M., Chopra I., Bhrdwaj A., Soni L., Shaheen U., Prajapati L., Sharma M., Sikarwar M.S. (2022). Structure-Based Virtual Screening, Molecular Docking, Molecular Dynamics Simulation and Pharmacokinetic Modelling of Cyclooxygenase-2 (COX-2) Inhibitor for the Clinical Treatment of Colorectal Cancer. Mol. Simul..

[81] Vitale P., Panella A., Scilimati A., Perrone M.G. (2016). COX-1 Inhibitors: Beyond Structure Toward Therapy. Med. Res. Rev..

[82] Blobaum A.L., Marnett L.J. (2007). Structural and Functional Basis of Cyclooxygenase Inhibition. J. Med. Chem..

[83] Kurumbail R.G., Stevens A.M., Gierse J.K., McDonald J.J., Stegeman R.A., Pak J.Y., Gildehaus D., Iyashiro J.M., Penning T.D., Seibert K. (1996). Structural Basis for Selective Inhibition of Cyclooxygenase-2 by Anti-Inflammatory Agents. Nature.

[84] Rådmark O.P. (2000). The Molecular Biology and Regulation of 5-Lipoxygenase. Am. J. Respir. Crit. Care Med..

[85] Ochs M.J., Suess B., Steinhilber D. (2014). 5-Lipoxygenase m RNA and Protein Isoforms. Basic Clin. Pharmacol. Toxicol..

[86] Recchiuti A., Serhan C.N. (2012). Pro-Resolving Lipid Mediators (SPMs) and Their Actions in Regulating miRNA in Novel Resolution Circuits in Inflammation. Front. Immunol..

[87] Charlier C., Michaux C. (2003). Dual Inhibition of Cyclooxygenase-2 (COX-2) and 5-Lipoxygenase (5-LOX) as a New Strategy to Provide Safer Non-Steroidal Anti-Inflammatory Drugs. Eur. J. Med. Chem..

[88] Jan M.S., Ahmad S., Hussain F., Ahmad A., Mahmood F., Rashid U., Abid O.-R., Ullah F., Ayaz M., Sadiq A. (2020). Design, Synthesis, in-Vitro, in-Vivo and in-Silico Studies of Pyrrolidine-2,5-Dione Derivatives as Multitarget Anti-Inflammatory Agents. Eur. J. Med. Chem..

[89] Gedawy E.M., Kassab A.E., El Kerdawy A.M. (2020). Design, Synthesis and Biological Evaluation of Novel Pyrazole Sulfonamide Derivatives as Dual COX-2/5-LOX Inhibitors. Eur. J. Med. Chem..

[90] Sisa M., Dvorakova M., Temml V., Jarosova V., Vanek T., Landa P. (2020). Synthesis, Inhibitory Activity and in Silico Docking of Dual COX/5-LOX Inhibitors with Quinone and Resorcinol Core. Eur. J. Med. Chem..

[91] Jacob J.P., Manju S.L. (2021). Novel Approach of Multi-Targeted Thiazoles and Thiazolidenes toward Anti-Inflammatory and Anticancer Therapy—Dual Inhibition of COX-2 and 5-LOX Enzymes. Med. Chem. Res..

[92] Ahmad S., Mahnashi M.H., Alyami B.A., Alqahtani Y.S., Ullah F., Ayaz M., Tariq M., Sadiq A., Rashid U. (2021). Synthesis of Michael Adducts as Key Building Blocks for Potential Analgesic Drugs: In Vitro, in Vivo and in Silico Explorations. Drug Des. Dev. Ther..

[93] Qandeel N.A., El-Damasy A.K., Sharawy M.H., Bayomi S.M., El-Gohary N.S. (2020). Synthesis, in Vivo Anti-Inflammatory, COX-1/COX-2 and 5-LOX Inhibitory Activities of New 2,3,4-Trisubstituted Thiophene Derivatives. Bioorg. Chem..

[94] Jacob P.J., Manju S.L. (2020). Identification and Development of Thiazole Leads as COX-2/5-LOX Inhibitors through in-Vitro and in-Vivo Biological Evaluation for Anti-Inflammatory Activity. Bioorg. Chem..

[95] Sadiq A., Mahnashi M.H., Alyami B.A., Alqahtani Y.S., Alqarni A.O., Rashid U. (2021). Tailoring the Substitution Pattern of Pyrrolidine-2,5-Dione for Discovery of New Structural Template for Dual COX/LOX Inhibition. Bioorg. Chem..

[96] El-Miligy M.M.M., Al-Kubeisi A.K., El-Zemity S.R., Nassra R.A., Abu-Serie M.M., Hazzaa A.A. (2021). Discovery of Small Molecule Acting as Multitarget Inhibitor of Colorectal Cancer by Simultaneous Blocking of the Key COX-2, 5-LOX and PIM-1 Kinase Enzymes. Bioorg. Chem..

[97] Bošković J., Ružić D., Čudina O., Nikolic K., Dobričić V. (2022). Design of Dual COX-2 and 5-LOX Inhibitors with Iron-Chelating Properties Using Structure-Based and Ligand-Based Methods. Lett. Drug Des. Discov..

[98] Da Cruz R.M.D., Mendonça-Junior F.J.B., De Mélo N.B., Scotti L., De Araújo R.S.A., De Almeida R.N., De Moura R.O. (2021). Thiophene-Based Compounds with Potential Anti-Inflammatory Activity. Pharmaceuticals.

[99] Francis P., Chakraborty K. (2021). Anti-Inflammatory Scalarane-Type Sesterterpenes, Erectascalaranes A–B, from the Marine Sponge Hyrtios Erectus Attenuate pro-Inflammatory Cyclooxygenase-2 and 5-Lipoxygenase. Med. Chem. Res..

[100] Mphahlele M.J., Onwu E.E., Agbo E.N., Maluleka M.M., More G.K., Choong Y.S. (2022). Synthesis, in Vitro and in Silico Enzyme (COX-1/2 & LOX-5), Free Radical Scavenging and Cytotoxicity Profiling of the 2,4-Dicarbo Substituted Quinazoline 3-Oxides. Med. Chem. Res..

[101] Javed M.A., Ashraf N., Saeed Jan M., Mahnashi M.H., Alqahtani Y.S., Alyami B.A., Alqarni A.O., Asiri Y.I., Ikram M., Sadiq A. (2021). Structural Modification, In Vitro, In Vivo, Ex Vivo, and In Silico Exploration of Pyrimidine and Pyrrolidine Cores for Targeting Enzymes Associated with Neuroinflammation and Cholinergic Deficit in Alzheimer’s Disease. ACS Chem. Neurosci..

[102] Saraf P., Nath Tripathi P., Kumar Tripathi M., Tripathi A., Verma H., Kumar Waiker D., Singh R., Kumar Shrivastava S. (2022). Novel 5,6-Diphenyl-1,2,4-Triazine-3-Thiol Derivatives as Dual COX-2/5-LOX Inhibitors Devoid of Cardiotoxicity. Bioorg. Chem..

[103] Bar F.M.A., Sameti M., Foudah A.I., Haque A., Elsbaey M. (2022). In Vitro and in Silico Inhibition of COX-2 and 5-LOX by Beta-Carboline Alkaloids from the Seeds of *Peganum harmala* L. S. Afr. J. Bot..

[104] Mahnashi M.H., Alshehri O.M. (2022). Isolation, In Vitro and In Silico Anti-Alzheimer and Anti-Inflammatory Studies on Phytosteroids from Aerial Parts of Fragaria × Ananassa Duch. Biomolecules.

[105] Javed M.A., Bibi S., Jan M.S., Ikram M., Zaidi A., Farooq U., Sadiq A., Rashid U. (2022). Diclofenac Derivatives as Concomitant Inhibitors of Cholinesterase, Monoamine Oxidase, Cyclooxygenase-2 and 5-Lipoxygenase for the Treatment of Alzheimer’s Disease: Synthesis, Pharmacology, Toxicity and Docking Studies. RSC Adv..

[106] Alqahtani Y.S., Jan M.S., Mahnashi M.H., Alyami B.A., Alqarni A.O., Rashid U., Mahmood F., Tariq M., Sadiq A. (2022). Anti-Inflammatory Potentials of β-Ketoester Derivatives of N-Ary Succinimides: In Vitro, In Vivo, and Molecular Docking Studies. J. Chem..

[107] Mahmood F., Khan J.A., Mahnashi M.H., Jan M.S., Javed M.A., Rashid U., Sadiq A., Hassan S.S.U., Bungau S. (2022). Anti-Inflammatory, Analgesic and Antioxidant Potential of New (2S,3S)-2-(4-Isopropylbenzyl)-2-Methyl-4-Nitro-3-Phenylbutanals and Their Corresponding Carboxylic Acids through In Vitro, In Silico and In Vivo Studies. Molecules.

[108] Nagesh K.M.J., Prashanth T., Khamees H.A., Khanum S.A. (2022). Synthesis, Analgesic, Anti-Inflammatory, COX/5-LOX Inhibition, Ulcerogenic Evaluation, and Docking Study of Benzimidazole Bearing Indole and Benzophenone Analogs. J. Mol. Struct..

[109] Bošković J., Dobričić V., Mihajlović M., Kotur-Stevuljević J., Čudina O. (2023). Synthesis, Evaluation of Enzyme Inhibition and Redox Properties of Potential Dual COX-2 and 5-LOX Inhibitors. Pharmaceuticals.

[110] Du L., Du S., Li J., Wang H. (2023). Design, Synthesis, and Biological Evaluation of Dual-Target COX-2/5-LOX Inhibitors for the Treatment of Inflammation. Med. Chem. Res..

[111] El-Miligy M.M.M., Al-Kubeisi A.K., Bekhit M.G., El-Zemity S.R., Nassra R.A., Hazzaa A.A. (2023). Towards Safer Anti-Inflammatory Therapy: Synthesis of New Thymol–Pyrazole Hybrids as Dual COX-2/5-LOX Inhibitors. J. Enzym. Inhib. Med. Chem..

[112] Badawi W.A., Rashed M., Nocentini A., Bonardi A., Abd-Alhaseeb M.M., Al-Rashood S.T., Veerakanellore G.B., Majrashi T.A., Elkaeed E.B., Elgendy B. (2023). Identification of New 4-(6-Oxopyridazin-1-Yl)Benzenesulfonamides as Multi-Target Anti-Inflammatory Agents Targeting Carbonic Anhydrase, COX-2 and 5-LOX Enzymes: Synthesis, Biological Evaluations and Modelling Insights. J. Enzym. Inhib. Med. Chem..

[113] Ragab M.A., Eldehna W.M., Nocentini A., Bonardi A., Okda H.E., Elgendy B., Ibrahim T.S., Abd-Alhaseeb M.M., Gratteri P., Supuran C.T. (2023). 4-(5-Amino-Pyrazol-1-Yl)Benzenesulfonamide Derivatives as Novel Multi-Target Anti-Inflammatory Agents Endowed with Inhibitory Activity against COX-2, 5-LOX and Carbonic Anhydrase: Design, Synthesis, and Biological Assessments. Eur. J. Med. Chem..

[114] Chen W., Xu Q., Ma X., Mo J., Lin G., He G., Chu Z., Li J. (2023). Synthesis and Biological Evaluation of N-(Benzene Sulfonyl)Acetamide Derivatives as Anti-Inflammatory and Analgesic Agents with COX-2/5-LOX/TRPV1 Multifunctional Inhibitory Activity. Bioorg. Med. Chem. Lett..

[115] Philoppes J.N., Abdelgawad M.A., Abourehab M.A.S., Sebak M., Darwish M.A., Lamie P.F. (2023). Novel *N*-Methylsulfonyl-Indole Derivatives: Biological Activity and COX-2/5-LOX Inhibitory Effect with Improved Gastro Protective Profile and Reduced Cardio Vascular Risks. J. Enzym. Inhib. Med. Chem..

[116] Coskun G.P., Ozhan Y., Dobričić V., Bošković J., Reis R., Sipahi H., Sahin Z., Demirayak S. (2023). Discovery of Novel Thiophene/Hydrazones: In Vitro and In Silico Studies against Pancreatic Cancer. Pharmaceutics.

[117] Soliman A.F., Abdel Bar F.M., Sallam A., Galala A.A. (2023). New Neuroprotective Sesquiterpene Lactate Esters from Carotol Biotransformation. S. Afr. J. Bot..

[118] Elgohary M.K., Abd El Hadi S.R., Abo-Ashour M.F., Abo-El Fetoh M.E., Afify H., Abdel-Aziz H.A., Abou-Seri S.M. (2023). Fragment Merging Approach for the Design of Thiazole/Thiazolidine Clubbed Pyrazoline Derivatives as Anti-Inflammatory Agents: Synthesis, Biopharmacological Evaluation and Molecular Modeling Studies. Bioorg. Chem..

[119] Qahtan M.Q.M., Bakhite E.A., Kumari J., Sayed A.M., Kandeel M., Sriram D., Abdu-Allah H.H.M. (2023). Synthesis, Biological Evaluation and Molecular Docking Study of Some New 4-Aminosalicylic Acid Derivatives as Anti-Inflammatory and Antimycobacterial Agents. Bioorg. Chem..

[120] El-Shiekh R.A., Shalabi A.A., Al-Hawshabi O.S.S., Ayman Salkini M., Abdel-Sattar E. (2023). Anticholinesterase and Anti-Inflammatory Constituents from Caralluma Awdeliana, a Medicinal Plant from Yemen. Steroids.

[121] Al-Wahaibi L.H., Abdel-Rahman M.H., El-Adl K., Youssif B.G.M., Bräse S., Abdel-Aziz S.A. (2024). New Diaryl-1,2,4-Triazolo[3,4-*a*]Pyrimidine Hybrids as Selective COX-2/sEH Dual Inhibitors with Potent Analgesic/Anti-Inflammatory and Cardioprotective Properties. ACS Omega.

[122] Chaaban I., Hafez H., Hazzaa A., Domiati S., Abd El Galil K.H., Hdeib F., Belal A.S.F., Ragab H. (2024). Experimental Investigation and Molecular Simulations of Quinone Related Compounds as COX/LOX Inhibitors. Inflammopharmacology.

[123] El-Miligy M.M.M., Al-Kubeisi A.K., Nassra R.A., El-Zemity S.R., Hazzaa A.A. (2024). Discovery of New Thymol-3,4-Disubstituted Thiazole Hybrids as Dual COX-2/5-LOX Inhibitors with in Vivo Proof. J. Enzym. Inhib. Med. Chem..

[124] Khadri M.J.N., Ramu R., Simha N.A., Khanum S.A. (2024). Synthesis, Molecular Docking, Analgesic, Anti-Inflammatory, and Ulcerogenic Evaluation of Thiophene-Pyrazole Candidates as COX, 5-LOX, and TNF-α Inhibitors. Inflammopharmacology.

[125] Mahnashi M.H., Rashid U., Almasoudi H.H., Nahari M.H., Ahmad I., Binshaya A.S., Abdulaziz O., Alsuwat M.A., Jan M.S., Sadiq A. (2024). Modification of 4-(4-Chlorothiophen-2-Yl)Thiazol-2-Amine Derivatives for the Treatment of Analgesia and Inflammation: Synthesis and in Vitro, in Vivo, and in Silico Studies. Front. Pharmacol..

[126] Akbar H.S., Muhammad N., Jan M.S., Zafar R., Ali S., Sadia H., Alomar T.S., AlMasoud N., Rauf A., Sharma R. (2024). Design, Synthesis, Molecular Docking, and Anti-Inflammatory Potential of Amide Coupling Carboxylate Derivatives. ChemistrySelect.

[127] Schrödinger L.L.C. (2013). MacroModel.

